# Variability in Action Selection Relates to Striatal Dopamine 2/3 Receptor Availability in Humans: A PET Neuroimaging Study Using Reinforcement Learning and Active Inference Models

**DOI:** 10.1093/cercor/bhz327

**Published:** 2020-02-21

**Authors:** Rick A Adams, Michael Moutoussis, Matthew M Nour, Tarik Dahoun, Declan Lewis, Benjamin Illingworth, Mattia Veronese, Christoph Mathys, Lieke de Boer, Marc Guitart-Masip, Karl J Friston, Oliver D Howes, Jonathan P Roiser

**Affiliations:** 1 Institute of Cognitive Neuroscience, University College London, London WC1N 3AZ, UK; 2 Division of Psychiatry, University College London, London W1T 7NF, UK; 3 Psychiatric Imaging Group, Robert Steiner MRI Unit, MRC London Institute of Medical Sciences, Hammersmith Hospital, London W12 0NN, UK; 4 Institute of Clinical Sciences, Faculty of Medicine, Imperial College London, Hammersmith Hospital, London W12 0NN, UK; 5 Wellcome Centre for Human Neuroimaging, University College London, London WC1N 3BG, UK; 6 Max Planck-UCL Centre for Computational Psychiatry and Ageing Research, London WC1B 5EH, UK; 7 Department of Psychosis Studies, Institute of Psychiatry, Psychology & Neuroscience (IoPPN), King’s College London, London SE5 8AF, UK; 8 Department of Psychiatry, University of Oxford, Warneford Hospital, Oxford OX3 7JX, UK; 9 Centre for Neuroimaging Sciences, Institute of Psychiatry, Psychology & Neuroscience (IoPPN), King's College London, London SE5 8AF, UK; 10 Scuola Internazionale Superiore di Studi Avanzati (SISSA), 34136 Trieste, Italy; 11 Translational Neuromodeling Unit (TNU), Institute for Biomedical Engineering, University of Zurich and ETH Zurich, 8032 Zurich, Switzerland; 12 Aging Research Center, Karolinska Institute, 171 65 Stockholm, Sweden

**Keywords:** active inference, action selection, decision temperature, dopamine 2/3 receptors, go no-go task, reinforcement learning

## Abstract

Choosing actions that result in advantageous outcomes is a fundamental function of nervous systems. All computational decision-making models contain a mechanism that controls the variability of (or confidence in) action selection, but its neural implementation is unclear—especially in humans. We investigated this mechanism using two influential decision-making frameworks: active inference (AI) and reinforcement learning (RL). In AI, the precision (inverse variance) of beliefs about policies controls action selection variability—similar to decision ‘noise’ parameters in RL—and is thought to be encoded by striatal dopamine signaling. We tested this hypothesis by administering a ‘go/no-go’ task to 75 healthy participants, and measuring striatal dopamine 2/3 receptor (D_2/3_R) availability in a subset (*n* = 25) using [^11^C]-(+)-PHNO positron emission tomography. In behavioral model comparison, RL performed best across the whole group but AI performed best in participants performing above chance levels. Limbic striatal D_2/3_R availability had linear relationships with AI policy precision (*P* = 0.029) as well as with RL irreducible decision ‘noise’ (*P* = 0.020), and this relationship with D_2/3_R availability was confirmed with a ‘decision stochasticity’ factor that aggregated across both models (*P* = 0.0006). These findings are consistent with occupancy of inhibitory striatal D_2/3_Rs decreasing the variability of action selection in humans.

## Introduction

To optimize behavior, the brain must choose actions that are expected to result in preferred outcomes. Active inference (AI) and reinforcement learning (RL) propose distinct computational mechanisms underpinning this fundamental ability, and assign differing roles to mesolimbic dopamine signaling. Common to both models is a mechanism controlling decision stochasticity, that is, variability in action selection. However, the neurobiological implementation of this mechanism is not well understood, especially in humans. Evidence in animal studies indicates that dopamine, acting at striatal dopamine 2 receptors in particular, modulates decision stochasticity, although numerous contradictory findings about the direction of this effect exist (see Discussion) (Cagniard et al. 2006; Beeler et al. 2010; Pesek-Cotton et al. 2011; Wunderlich et al. 2012; Stopper et al. 2013; Eisenegger et al. 2014; Kwak et al. 2014; Costa et al. 2015; Lee et al. 2015). One functional magnetic resonance imaging (fMRI) study in humans reported that a dynamically changing decision stochasticity variable correlated with activation in the midbrain, which contains dopamine neurons (Schwartenbeck et al. 2015); however, fMRI cannot measure dopamine directly.

In this study, we used both the AI and RL computational decision-making frameworks to illuminate the contribution of striatal dopamine 2/3 receptors (D_2/3_Rs) to decision stochasticity and action biases, using in vivo neuroimaging with positron emission tomography (PET). However, PET measurement of receptor availability occurs over 30–60 min (Egerton et al. 2010), and so cannot be used to assess dopamine activity on a timescale of single trials. Here, we use it to index dopamine D_2/3_R availability, which can be used as an indirect measure of tonic dopamine levels (Caravaggio et al. 2016). We used two modeling frameworks primarily to check our findings were robust—that is, to assess whether dopamine receptor availability correlated with parameters governing decision stochasticity in both frameworks. A secondary aim was to compare the frameworks’ performance in modeling empirical choices, as this has not been done before. We first briefly explain and compare the two frameworks; specifically, how they solve the computational problem of optimizing action selection to obtain reward and avoid punishment. We then examine the respective roles proposed by AI and RL for dopamine signaling in these models (for details of the models, see Methods).

### A Comparison of Reinforcement Learning and Active Inference

RL and AI both provide accounts of how the brain approximates Bayesian reasoning (i.e., the optimal use of all available information), yet the algorithmic solutions each postulates are different. RL proposes that agents perform actions to maximize expected cumulative future reward. Standard ‘model-free’ RL algorithms (e.g., Rescorla–Wagner, as used here) propose that agents achieve this by learning state-action values during direct trial-and-error experience of reward prediction errors (Sutton and Barto 1998), and then using those values to guide action selection. More sophisticated ‘model-based’ RL algorithms (Daw et al. 2005, 2011) use additional information about the transition structure of the environment to infer the current state (and its uncertainty) and to plan future actions; some also incorporate uncertainty about action outcomes themselves (Daw et al. 2006).

In contrast, AI is a fully Bayesian scheme that assumes both perception and action obey the same principle: the minimization of surprise (i.e., prediction errors). Thus, AI models of decision-making (formally, Markov Decision Processes – MDPs; Friston et al. 2013) combine inference about states of the world together with action planning and selection (which are usually treated separately in RL) into a single model (Fig. S1*A*). Rather than maximizing long-term reward, an agent is endowed with prior preferences, termed ‘beliefs,’ about its goal states (e.g., that it will be fed, hydrated, warm, etc). It then samples actions that minimize the difference between predicted and preferred outcomes. The quintessential distinction between AI and model-free RL rests upon the difference between inference and learning. Thus, an AI agent infers the current context given a cue (e.g., ‘this banana is sweet because it is yellow’) and then infers what it is going to do to fulfill prior preferences (e.g., ‘I am very likely to eat this banana, because I prefer sweet things’). In contrast, a model-free RL agent might choose to eat the banana because eating yellow bananas has been rewarding in the past.

In this regard—and in the simple task employed in the present study—AI is similar to ‘planning as inference’ algorithms (Attias 2003; Botvinick and Toussaint 2012; Gershman 2019), which infer actions from a joint distribution of actions, states, and rewards, given an agent’s expectation that it will maximize reward (or in AI terms, the similarity to its goal states). One difference is that although ‘planning as inference’ (and model-based RL) algorithms update their confidence about current states, they do not generally update their confidence about action selection: AI does both, enabling optimal, risk-sensitive behavior.

**Figure 1:**
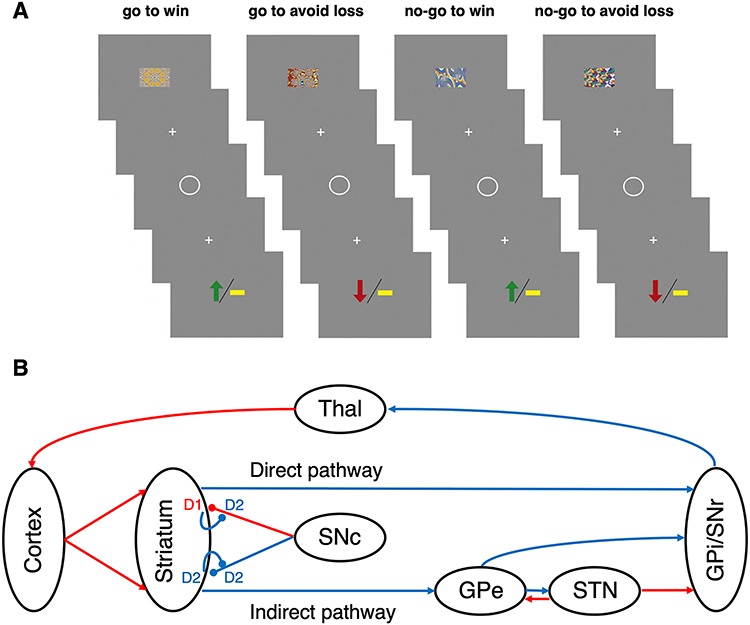
The go no-go task and the corticostriatal circuitry it explores (*A*). A schematic illustrating the go no-go task. The participant sees one of four fractal images for 1000 ms, followed by a fixation cross for 250–2000 ms, then a circle during which he/she must make (or not make) a button press response within 800 ms, followed by another fixation cross for 1000 ms, and then the outcome (loss, no-change, or win). See the text for details. (*B*) Corticostriatal circuitry and dopamine receptors. Excitatory connections are red arrows, and inhibitory connections are blue arrows. Modulatory (dopaminergic) connections end in balls: this can be excitatory (via D_1_Rs) or inhibitory (via D_2_Rs). The short connections leading back from the striatum to the dopaminergic pathways depict autoreceptor (D_2_R) effects. The direct pathway is excitatory overall, the indirect pathway inhibitory. Dopamine excites the former and inhibits the latter, thus increasing activity in both pathways. D_1_Rs are less sensitive to small dopamine concentrations than D_2_Rs, meaning that phasic bursts are best detected by D_1_Rs, and dips in tonic firing by D_2_Rs (Dreyer et al. 2010). An influential RL model of striatal function (Schultz et al. 1997; Frank et al. 2004) proposed that positive reward prediction errors are signaled by phasic bursting of dopamine neurons, activating D_1_Rs and increasing synaptic plasticity in the direct pathway, thus increasing the probability that the recent action would be repeated (‘go learning’); whereas negative reward prediction errors would be signaled by dips in tonic dopamine activity, lowering D_2_R inhibition of the inhibitory indirect pathway and thus decreasing the probability the recent action would be repeated (‘no-go learning’). In addition, the interactions between GPe and STN in the indirect pathway may serve to increase stochasticity (Sridharan et al. 2006), that is, vary the dominant pathway, aided by the extensive lateral competition within the pathways parallel circuits (Keeler et al. 2014; Burke et al. 2017).

To investigate the computational nature and neural implementation of choice stochasticity, we used the ‘orthogonalized go/no-go’ task (Guitart-Masip et al. 2012) and compared AI with Rescorla–Wagner models, the best established RL accounts of this task. In this task, participants must learn whether to make (go) or withhold (no-go) a button press in response to four visual stimuli (defining unique ‘contexts’; Fig. 1*A*), in order to either win money (positive valence contexts) or avoid losing money (negative valence contexts). Crucially, this task decorrelates the optimal action from the valence of the context, and can thus demonstrate the (here sometimes suboptimal) tendency to go (rather than no-go) to obtain reward, and no-go (rather than go) to avoid punishment—known as ‘Pavlovian biases.’ Rescorla–Wagner RL models that include Pavlovian bias parameters can explain such behavioral biases well (Guitart-Masip et al. 2012, 2014; Cavanagh et al. 2013; Chowdhury et al. 2013; Swart et al. 2017). However, several open questions remain, including:

(i) how best to model (apparent) randomness in choice behavior (and its relation to dopamine);
(ii) how the Pavlovian biases described above emerge (also see Moutoussis et al. 2018; de Boer et al. 2019); and
(iii) whether and how participants deploy knowledge about the task structure to optimize behavior.

**Figure 2:**
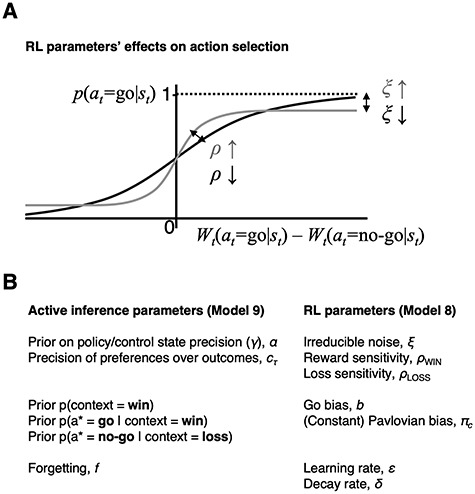
Action selection in RL, and how the RL parameters relate to those of AI. (*A*) This illustrates the effects of some RL parameters on action choices, but NB this is not how $\rho$ was implemented in the model itself. The point is that the effect of $\rho$ on decision making is mathematically identical to that of a softmax inverse temperature parameter, which scales the gradient of this plot, and hence how deterministically differences in action weights translate into actions. $\xi$ scales the distance of the asymptote from 1 or 0, that is, a consistent level of stochasticity no matter how great the difference between action weights. The role of $\gamma$ in the AI model is most similar to that of $\rho$ here, except that it is updated on every trial, so as confidence increases, actions become more deterministic. (*B*) The parameters are listed for AI and the most complex RL model. The parameters are separated into three broad groups across both models: those pertaining to stochasticity, biases, and the speed of belief changes (respectively).

Rescorla–Wagner and AI offer different answers to these questions; hence, a direct comparison of the frameworks is useful (Fig. 2).

(i) Randomness in choice behavior: In the AI scheme implemented here (Figs S1 and S2), the ‘precision over policies’ ($\gamma$) controls how confidently an agent selects the most probable policy (action). As this is a Bayesian model, the agent has a prior over this precision ($\alpha$) which is updated—that is, optimized—in light of its experience. In RL, choice variability is governed by the ‘inverse temperature’ parameter of a softmax response function, or equivalently—as in the Rescorla–Wagner models implemented here (Figs 2*A* and S1*C*)—by an ‘outcome sensitivity’ parameter ($\rho$), and also an ‘irreducible noise’ parameter ($\xi$) that allows actions to be taken even when their values are exceptionally low (e.g., due to attentional lapses). Crucially, in AI, $\gamma$ is optimized as the agent becomes more confident about what it should do in a given context; whereas in RL, the $\rho$ and $\xi$ parameters are typically fixed throughout.
(ii) Pavlovian biases: In Rescorla–Wagner RL models of this task, response biases emerge from parameters that drive valence-dependent biases in action selection or learning. Specifically, the ‘Pavlovian bias’ parameter, $\pi$, promotes go and no-go actions in positively and negatively valanced states, respectively. In the AI model, by contrast, we encoded these biases as prior beliefs about contingency; for example, that if the context is one of opportunity (i.e., reward), the best action is go: $p\!\left({a}^{\ast }=\mathrm{go}|\mathrm{context}=\mathrm{W}\right)$. If such biases are beliefs, rather than fixed action-selection biases, they may be easier to overcome within the task and may be updated upon repeating the task (Moutoussis et al. 2018).
(iii) Knowledge about task structure: In AI models, updates to beliefs about the context to which the visual stimulus belongs are Bayes optimal; that is, proportional to uncertainty. In this simple task, this is similar to what a ‘model-based’ or ‘planning as inference’ RL agent would do. However, the AI scheme used here also assumes that beliefs about recent states are stored in working memory, and are thus vulnerable to decay back toward their initial (i.e., prior) values. This decay depends on how many trials elapse before the same stimulus is encountered again; weighted by a ‘forgetting’ parameter, $f$. In contrast, in the Rescorla–Wagner RL models used here, the size of value-updates is determined by a fixed ‘learning rate’ parameter, $\varepsilon$, which is insensitive to uncertainty. One RL model also incorporates the forgetting of action values, due to a decay parameter $\delta$. See Figure 2*B* for a comparison of the parameters from both models.

### The Role of Dopaminergic Signaling in Reinforcement Learning and Active Inference

AI and RL postulate differing computational roles for striatal dopaminergic signaling. The process theory behind AI proposes that mesostriatal dopaminergic projections encode precision over policies; in particular, that tonic dopamine activity encodes a “prior on policy precision parameter,” *α*, while phasic firing reflects updates to this prior to form a posterior policy precision, $\gamma$ (Friston et al. 2013; FitzGerald et al. 2015; Schwartenbeck et al. 2015). In contrast, influential RL theories propose that tonic dopamine activity encodes the expected average rate of reward (Niv et al. 2007)—and thus affects response vigor, rather than choice stochasticity—while phasic firing encodes a temporal difference reward prediction error (Schultz et al. 1997) (although see Sharpe and Schoenbaum 2018).

To test dopamine’s relationship to choice stochasticity, in a subset of our sample, we used the D_2/3_R agonist PET ligand [^11^C]-(+)-4-propyl-9-hydroxynaphthoxazine ([^11^C]-(+)-PHNO) to measure striatal D_2/3_R availability (BP_ND_), and investigated the relationship between this dopaminergic measure and the relevant parameters from both models.

For the AI model, we hypothesized that tonic striatal dopamine, which activates D_2/3_Rs (Dreyer et al. 2010), encodes an agent’s prior on precision over policies, $\alpha$. As [^11^C]-(+)-PHNO competes with endogenous dopamine to bind to D_2/3_Rs, its BP_ND_ is negatively related to synaptic dopamine concentration (Caravaggio et al. 2016). Therefore, we predicted a negative correlation between BP_ND_ and the prior on policy precision parameter, $\alpha$. Given that in RL the reward and punishment sensitivity and irreducible noise parameters, ${\rho}$_win_, ${\rho}$_loss_, and $\xi$, determine choice randomness, we also predicted negative relationships between BP_ND_ and ${\rho}$_win_, ${\rho}$_loss_ and the irreducible noise parameter, $\xi$. However, these relationships might also contain a quadratic element (as seen in prior studies examining the relationship between both no-go and reversal learning and D_2/3_R availability as measured by PET; Groman et al. 2011; Cox et al. 2015)—see the Discussion for more on this point.

### Study Aims

We addressed two key questions:

(i) Is there evidence that one or more parameters governing variability in action selection (the prior on policy precision, $\alpha$ (in AI), or $\rho$ and $\xi$ parameters (in RL)) are encoded by transmission at striatal D_2/3_Rs?
(ii) Does either AI or any previously employed Rescorla–Wagner RL model better explain the behavior of healthy participants on an orthogonalized go/no-go task?

## Methods and Materials

### Participants

The study was approved by the local NHS Research Ethics Committee (Ref. 15/LO/0011) and the Administration of Radioactive Substances Advisory Committee (Ref. 630/3764/32523), and was conducted at Imanova Centre for Imaging Sciences, London and the Institute of Cognitive Neuroscience, UCL. 75 healthy volunteers (mean age 26.8 years [std 7.5], 40 male) with no history of neurological or psychiatric illness were recruited from the ICN participant database and underwent behavioral testing in the ICN. Questionnaire measures of IQ (the Wechsler Test of Adult Reading, WTAR; Wechsler 2001) and working memory (Digit Span) were also administered to all participants: mean IQ was 106.3 [std 8.2], mean Digit Span was 17.0 [std 4.1]. A subset of 26 of these participants (mean age 27.5 years [std 8.5], 10 male) also had [^11^C]-(+)-PHNO PET imaging at Imanova within up to 15 days (mean 6.0 days [std 5.5]) of behavioral testing. One PET participant had to terminate their scan due to nausea. All participants provided written informed consent.

### Behavioral Task and Behavioral Analysis

The task (Fig. 1*A*) was the learning version of the orthogonalized go no-go task (Guitart-Masip et al. 2012). In this version, only 36 trials per condition were used instead of 60, due to time constraints. The participants were instructed that they would see one of four fractals in each trial, each corresponding to a different trial type, and that they should respond after seeing the white circle: the correct response would be either making or withholding a button press. They were also instructed that they had to learn through trial and error what response to make, and that in some conditions they could win 10 pence (green upward arrow) or get nothing (yellow bar), and in others they could lose 10 pence (red downward arrow) or get nothing (yellow bar). They were also informed about the probabilistic nature of the task, that is, that correct responses would result in the best outcome 80% of the time, and that the contingencies would not change during the task. Prior to the learning task they performed a practice session, to familiarize them with timing requirements: they had to respond within 800 ms, if they were to do so; and between 500 and 800 ms, responses were counted as go but participants saw “your response was too slow” on the screen. During the task, the trial types were randomly permuted, and for each participant the fractals were randomly allocated to the conditions. Participants were paid their total winnings (if above zero) along with a standard participation fee at the end of the experiment.

We devised some simple behavioral measures—‘normalized switches’ and ‘trials to decision point’ that we hypothesized would differ between participants who were better fit by AI or by RL. ‘Normalized switches’ was simply the mean proportion of all trials (across contexts) when subjects changed their responses. The ‘decision point’ was defined as the point that maximized a participant’s response ‘consistency’ if it was assumed she made her final decision about the context after that trial (Equation 1). If several trials fulfilled this criterion, the earliest was chosen. Here, ‘consistency’ was the extent to which participants chose sequences of identical responses—irrespective of being correct or incorrect—allowing for the possibility that they might change their minds up to once.

(1) \begin{equation*} {\displaystyle \begin{array}{l}\mathrm{Actions},\kern0.5em a\in \left[-1,1\right]\\{}\mathrm{Consistency},\mathrm{con}=\left|\sum \left({a}_1,\dots, {a}_d\right)\right|+\left|\sum \left({a}_{d+1},\dots, {a}_{36}\right)\right|\\{}\mathrm{Decision}\ \mathrm{point},\kern0.5em d=\underset{d\in \left[1,\dots, 36\right]}{\arg \max \left(\mathrm{con}\right)}\end{array}} \end{equation*}

Thus, participants choosing [–1 –1 –1 –1 –1 –1 –1 –1] and [1 1 1 –1 –1 –1 –1 –1] are both maximally consistent (1), whereas [1 –1 –1 1 1 1 –1 1] is more inconsistent. Decision points would be trials 0, 3, and 3 in these examples. Mediation analyses of the relationships between consistency or trials to decision point and model evidence were performed using the Variational Bayesian Analysis toolbox (Daunizeau et al. 2014).

### Computational Modeling – Reinforcement Learning

#### Reinforcement Learning Models

The RL models were based on those used previously (Guitart-Masip et al. 2012; Cavanagh et al. 2013). These models compute the probability of taking an action $a$ at time $t$ given one is in state *s* as a function of the weight the model has assigned to taking that action in that state $W\left({a}_t|{s}_t\right)$ versus any other action $\sum_{a^{\prime }}W\left(a{\prime}_t|{s}_t\right)$. This was done by subjecting the action weights to a compressed softmax function (Sutton and Barto 1998), which includes ‘irreducible noise’ $\xi$ (Equation 2)—that is, a certain level of stochasticity in decision-making no matter how good one action seems compared with the others. There is no inverse temperature parameter—instead, the model uses a reward/punishment sensitivity parameter $\rho$ (Equation 3), which is functionally identical:

(2) \begin{equation*} p\!\left({a}_t\left|\kern0.1em {s}_t\right.\right)=\xi \left[\frac{\exp \left(W\left({a}_t\left|\kern0.1em {s}_t\right.\right)\right)}{\sum \limits_d\exp \left(W\left({a}^{\prime}\left|\kern0.1em {s}_t\right.\right)\right)}\right]+\frac{1-\xi }{2} \end{equation*}

**Figure 3:**
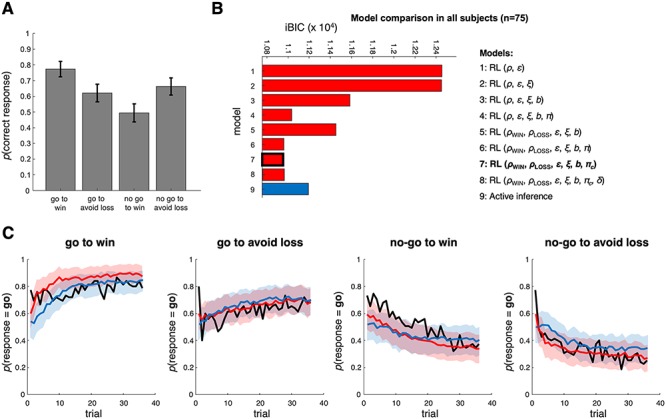
Performance and model comparison across all participants (*n* = 75). (*A*) The probability of making a correct response in each condition across all participants and trials. Participants do best in the ‘Pavlovian’ conditions, go to win and no-go to avoid loss. Error bars depict the standard error of the mean. (*B*) Model comparison across all participants using the integrated BIC (see text). Model 7 is the best model; AI is fifth. The RL model parameters shown here included: reward and loss sensitivities ${\rho}$_win_ and ${\rho}$_loss_, learning rate $\varepsilon$, irreducible noise $\xi$, go bias $b$, different forms of Pavlovian bias (see Methods), $\pi$ and ${\pi}_c$, and decay rate $\delta$. The AI model parameters are listed in Table 1 and Figure 2*B*. (C) These plots compare the actual probability of participants’ go responses (group averaged data) with model predictions in each condition. The probability of go responses is plotted in black. To create the model predictions, each model simulated responses using each participant’s estimated parameters 20 times, that is, 1500 times in total. The responses were then averaged and plotted in red (RL – Model 7) and blue (AI – Model 9) lines. 95% confidence intervals for the means of the simulations (shaded areas) were derived from the distributions of means of 100 samples of *n* = 75 participants.

Computation of the action weights differed between the different RL models (all listed in Fig. 3). The simplest models (1 and 2) used standard Rescorla–Wagner equations to update the $Q$ values of actions in states, with reward/punishment sensitivity $\rho$ and learning rate $\varepsilon$:

(3) \begin{equation*} {Q}_t\left({a}_t,{s}_t\right)={Q}_{t-1}\left({a}_t,{s}_t\right)+\varepsilon \left(\rho{r}_t-{Q}_{t-1}\left({a}_t,{s}_t\right)\right). \end{equation*}

The more sophisticated RL models added in extra parameters. Model 3 introduced a bias term *b* (Equation 4) that increased the weight for go actions:

(4) \begin{equation*} {\displaystyle \begin{array}{l}a=\mathrm{go}:\\{}{W}_t\left(a,s\right)={Q}_t\left(a,s\right)+b\\{}a=\mathrm{no}\hbox{-} \mathrm{go}:\\{}{W}_t\left(a,s\right)={Q}_t\left(a,s\right)\end{array}} \end{equation*}

Model 4 introduced a Pavlovian bias parameter $\pi$ (Equation 5) that increased the probability of a go action if the value of the current state was positive and decreased it if it was negative, thus making ‘go to win’ and ‘no-go to avoid loss’ more likely. This value was computed using Rescorla–Wagner updating:

(5) \begin{equation*} {\displaystyle \begin{array}{l}a=\mathrm{go}:\\{}{W}_t\left(a,s\right)={Q}_t\left(a,s\right)+b+\pi{V}_t(s)\\{}a=\mathrm{no}\hbox{-} \mathrm{go}:\\{}{W}_t\left(a,s\right)={Q}_t\left(a,s\right)\end{array}} \end{equation*}

(6) \begin{equation*} {V}_t\left({s}_t\right)={V}_{t-1}\left({s}_t\right)+\varepsilon \left(\rho{r}_t-{V}_{t-1}\left({s}_t\right)\right) \end{equation*}

Models 5 and 6 equipped Models 3 and 4 with separate sensitivities to reward and punishment, that is, ${\rho}$_win_ and ${\rho}$_loss_ respectively, via Equation 6. These parameters play an identical role to a softmax decision inverse temperature parameter (illustrated schematically in Fig. 2*A*). Model 7 used a ‘constant’ Pavlovian bias ${\pi}_c$, which—instead of being multiplied by the value of the state—was simply multiplied by a value of 1 (for rewards) or –1 (for punishments), from the first time the participant encountered one of these in that state. In other words, ${\pi}_c=\pi \cdot \operatorname{sgn}\left(V(s)\right)$. Model 7 is written out in full in Figure S1*C*.

Finally, Model 8 incorporated an additional ‘forgetting’ parameter. This was to discover whether adding this mechanism to the hitherto best-performing RL model could make it perform similarly to the AI model, which also contained a forgetting process. The forgetting parameter was decay rate $\delta$: on each trial, the $Q$ values of all unchosen actions depreciated toward zero by this constant factor (as in de Boer et al. 2017).

#### Model Fitting

Model parameters were estimated by transforming parameters so that they could, in transformed space, be described by normal distributions and then using expectation-maximization as previously described (Huys et al. 2011; Guitart-Masip et al. 2012).

#### Model Comparison

Models were compared using the integrated Bayesian Information Criterion (iBIC), as used previously (Huys et al. 2011; Guitart-Masip et al. 2012). In the case of the AI model, the model-fitting procedure calculated both the free-energy approximation to the log model evidence $F$ and the iBIC. As might be expected by the fact that both are estimates of the model evidence, the two were very closely correlated (*r* = 0.994, *P* = 10^−70^).

The iBIC approximates the log model evidence $\ln p\!\left(y|m\right)$ not by using the log likelihood (under the maximum-likelihood parameter estimates) for each individual, as in the standard BIC, but by weighting the likelihood by the posterior probability that the corresponding parameters obtain, given the ML estimate of the group parameters ${\hat{\theta}}$^ml^. This integral is approximated by sampling from the posterior distributions over the parameters $h$ a total of *N* = 2000 times per participant for $i$ participants:

(7) \begin{align*} 1\mathrm{n}\ p(y|{{{}^{{}^{{}^{\frown}}}}\kern-6pt{\theta}}^{\textrm{ML}})=&\;\sum \limits_i1\mathrm{n} \int p({y}_i|h)p(h|{{{}^{{}^{{}^{\frown}}}}\kern-6pt{\theta}}^{\textrm{ML}}) dh\nonumber\\ \simeq &\; \sum \limits_i1\mathrm{n}\frac{i}{N}\sum \limits_{n=1}^Np({y}_i|{h}_n) \end{align*}

The iBIC can then be computed (Equation 8). Smaller iBIC values indicate a better model: comparing iBIC values is akin with a Bayes Factor.

(8) \begin{align*} 1\mathrm{n}\ p\!\left(y\left|m\right.\right)=&\;1\mathrm{n}\int p\!\left(y\left|\theta \right.\right)p\!\left(\theta \left|m\right.\right) d\theta \nonumber\\ \simeq &\; \mathrm{iBIC}=-2\kern0.5em 1\mathrm{n}\ p\!\left(y\left|{{{}^{{}^{{}^{\frown}}}}\kern-6pt{\theta}}^{\textrm{ML}}\right.\right)+\left|\theta \right|1\mathrm{n}\left|s\right|\end{align*}

We also computed the mean pseudo *R*^2^ (Camerer and Ho 1999) across participants as a measure of the degree to which a given model performs better than chance (0 (or below) is at chance (or worse), and 1 is perfect). For $t$ trials, each having a probability of 0.5 of being correctly predicted by chance, the pseudo *R*^2^ is:

(9) \begin{equation*} \mathrm{pseudoR}2=\left|\frac{1\mathrm{n}\ p\!\left(y\left|{{{}^{{}^{{}^{\frown}}}}\kern-6pt{\theta}}^{\textrm{ML}}\right.\right)+1\mathrm{n}\left({0.5}^t\right)}{1\mathrm{n}\left({0.5}^t\right)}\right| \end{equation*}

### Computational Modeling – Active Inference

AI agents infer behavior using MDPs (Fig. S1*A*) with action-dependent state transitions. In an MDP, different states of the world can (probabilistically) lead to different outcomes (e.g., rewards), and an agent’s actions affect transitions from one state to others. The agent must infer both (i) what state it is in, given what it has done and observed so far (this is a partially observable MDP, or POMDP), and (ii) the best actions to take (or ‘policies’) given its current state and its prior preferences (Friston et al. 2013). The ‘best’ actions lead to (preferred) states or outcomes that have high a priori probability; see, planning as inference (Attias 2003; Botvinick and Toussaint 2012).

Essentially, the model (Fig. S1*A*) considers the observations initial, lose, null and win – $o=\left\{\mathrm{init},-1,0,1\right\}$ – in series $\tilde{o}=\left\{{o}_0,\dots, {o}_T\right\}$. These depend solely upon hidden states $\tilde{s}$. Transitions between the hidden states are determined by control states $u=\left\{\mathrm{Go},\mathrm{NoGo}\right\}$, sequences of which constitute policies . Actions are sampled from posterior beliefs over policies, and these beliefs have a precision $\gamma$. We now describe each of these variables in more detail, but please see Friston et al. (2013) for a comprehensive description of the model:

#### Prior Beliefs About States and Preferences

In the go no-go task, each visual stimulus belongs to one of four ‘contexts’ (go to win, go to avoid loss, no-go to win, no-go to avoid loss; Fig. 1*A*), which do not change. For each of the four contexts, we consider four states: an initial state upon seeing the stimulus, and three outcomes—lose, no-change and win: $\left\{s\right\}=\left[\mathrm{initial},\mathrm{lose},\mathrm{null},\scriptsize{\rm WIN}\right]\times \left[\mathrm{G}2\mathrm{W},\mathrm{G}2\mathrm{AL},\mathrm{NG}2\mathrm{W},\mathrm{NG}2\mathrm{AL}\right]$. This means there are 16 possible states; although in the task itself, only 12 are used—one initial state and two action-dependent outcomes for each of the four contexts (associated with each fractal cue).

Given the current stimulus, the agent infers which context it is currently in terms of a distribution over contexts $D\!\left(\mathrm{ctxt}\right)$. At the start of the task, this belief distribution $D\!\left({\mathrm{ctxt}}_{t=0}\right)$ is determined by the agent’s prior beliefs about the relative frequency of the four contexts—that are updated with repeated exposure to each stimulus. These beliefs are the following ‘Pavlovian’ priors ${P}_0$: the probability a context is one in which winning money is possible, the probability the best action is go given money can be won, and the probability the best action is no-go in a context where one must avoid loss: ${P}_0=\{p\!\left(\mathrm{context}=\mathrm{W}\right),p\!\left({a}^{\ast }=\mathrm{go}|\mathrm{context}=\mathrm{W}\right),p({a}^{\ast }=\mathrm{no}\ \mathrm{go}|\mathrm{context} = \mathrm{AL})\}$. (These priors are Pavlovian in the sense that the relative frequency of the contexts determines how likely an agent is to assume go is the correct action in a win context, etc). An example set of priors and the resulting $D\!\left({\mathrm{ctxt}}_{t=0}\right)$ is shown in Figure S2*A*.

The agent also has prior beliefs about outcomes, or preferences. We model agents’ preferences with a softmax function of objective returns $r$ at the outcome time $\sigma \left(r\left({s}_T\right)\!;{c}_{\tau}\right)$. The (inverse) temperature parameter ${c}_{\tau }$—termed the precision of prior preferences—describes how sensitive agents are to differences in returns (also see Fig. S2*B*). Thus:

(10) \begin{equation*} p\!\left({s}_T\left|m\right.\right)\infty \sigma\! \left(r\left({s}_T\right)\!;{c}_{\tau}\right) \end{equation*}

This describes a probability distribution over states ${s}_T$ at time $T$ (given the model $m$), which depends upon the returns associated with each state. We also hypothesized that outcome desirability is influenced by beliefs about the prevalence of the context in which it is obtained (in developing the model, this considerably improved the quality of model fits: unpublished data). Thus, agents’ beliefs temper their desires before they consider the difference their policies may make in attaining them. In this case, the agent’s prior preferences $\mathbf{c}$ are the product of the distribution over contexts and the subjective preferences for the states in those contexts (an example is illustrated in Fig. S2*B*):

(11) \begin{equation*} \mathrm{c}:= p\!\left({s}_T\left|m\right.\right)=D\!\left({\mathrm{ctxt}}_{t=0}\right)\sigma\! \left(r\left({s}_T\right)\!;{c}_{\tau}\right) \end{equation*}

#### States and Transitions

Transition matrices $\mathbf{B}\left\{\mathrm{go}\right\}$ and $\mathbf{B}\left\{\mathrm{nogo}\right\}$ represent the dependence of state transitions on policies or control states $\tilde{u}$: they contain the probabilities $p\!\left({s}_{t+1}|{s}_t,\pi \right)$ (Fig. S2*C*). They map ‘from’ states (listed in columns) ‘to’ states (listed in rows), given a policy, so for matrix $\mathbf{B}\left\{\mathrm{go}\right\}$, entry ${\mathbf{B}}_{ij}$ is the probability of transitioning from state $j$ (here, the initial state) to state $i$ (e.g., winning) if the go action is performed (i.e., *P* = 0.8 for the G2W condition). Here, we consider policies operating upon the states of a single trial (rather than a whole set of trials). As stimuli belong to fixed contexts, $\mathbf{B}$ is block-diagonal. Within each block, the initial state leads to the optimal outcome with probability 0.8, and to the suboptimal outcome with probability 0.2. Actions have no effect on ‘outcome states’ (win, lose, or no-change in all contexts), which hence map trivially onto themselves (Fig. S2*C*).

The task is conceptualized as a chain of single trials, or mini-games, for each stimulus, as follows:

1. Within the chain for each particular stimulus, the posterior probability about the context to which the stimulus belongs (i.e., the belief distribution over states) becomes the prior probability of the next trial in the same chain. Recently acquired evidence is subject to forgetting, however:
2. The chains affect each other only in that new information about each stimulus has to be kept in working memory while other, intervening trials involving different stimuli occur. The number of these intervening trials is ${n}_{\mathrm{gap}}-1$ (i.e., for consecutive trials in a chain, ${n}_{\mathrm{gap}}$ is 1), and the index of each trial in a chain is $t$. The forgetting parameter $f$ quantifies how leaky working memory is. We write:

(12) \begin{align*} D\!\left({\mathrm{ctxt}}_t\right)=&\;D\!\left({\mathrm{ctxt}}_{t-{n}_{\mathrm{gap}}}\right)\left(1-{f}^{n_{\mathrm{gap}}}\right)\nonumber\\ &+D\!\left({\mathrm{ctxt}}_{t=0}\right)\left(1-{\left(1-f\right)}^{n_{\mathrm{gap}}}\right) \end{align*}

$D\!\left({\mathrm{ctxt}}_{t=0}\right)$ is determined by the (Pavlovian) prior beliefs over the contexts (see above). Thus, over time, a participant’s beliefs about the prevalence of the contexts given the previous outcome in that context decays away from $D\!\left({\mathrm{ctxt}}_{t-n\mathrm{gap}}\right)$ and back toward $D\!\left({\mathrm{ctxt}}_{t=0}\right)$ until that context is revisited, ${n}_{\mathrm{gap}}$ trials later (illustrated with respect to getting a reward in the state $\mathrm{NG}2\mathrm{W}$ in Fig. S2*D*). Once the agent returns to that context, its (decayed) beliefs given the previous outcome $D\!\left({\mathrm{ctxt}}_t\right)$ are incorporated into its ‘stored’ posterior beliefs about the context, which do not decay.

Note that in this task, $\mathbf{B}$ is formulated, so that the mapping from states to outcomes $p\!\left(\tilde{o}|\tilde{s},m\right)$—normally contained in the $\mathbf{A}$ (likelihood) matrix—is deterministic, so no $\mathbf{A}$ matrix is required. This simplifies the model but is formally equivalent to the typical partially observable MDP notation incorporating both $\mathbf{B}$ and $\mathbf{A}$. The key point is that AI assumes that subjects use the information in the task instructions to construct a state space that they can use to do inference. This assumption determines the state space we use here.

#### Policy Choice

Assume that an agent believes that at time *t* they occupy a state ${s}_t$. They then need to choose a policy $\pi$ comprising a sequence of control states $\tilde{u}=\left\{{u}_0,\dots, {u}_T\right\}$ that leads to a desired outcome distribution $p\!\left({s}_T|m\right)$, abbreviated to $\mathbf{c}$ (Fig. S2*B*). Here, there is only one meaningful action; that is, no sequence, so $\tilde{u}$ is just go or no-go (we therefore use $\pi$ instead of $\tilde{u}$ throughout for reasons of clarity). If $\pi$ leads to a distribution over final or outcome states $p\!\left({s}_T|{s}_t,\pi \right)$—in this one timestep task, equivalent to $\mathbf{B}$—then success can be measured by the Kullback–Leibler divergence (in basic terms, the difference) between the anticipated and desired distributions, abbreviated to $\mathbf{Q}$. The agent can then choose policies according to this measure of their likely success. We can express this formally as follows (Friston et al. 2013):

(13) \begin{align*} &\mathbf{Q}:= -{D}_{\mathrm{KL}} \! \left[p\!\left({s}_T\left|{s}_t,\pi \right.\right)\!\left\Vert p\!\left({s}_T\left|m\right.\right)\right.\!\!\right] \nonumber\\ & {\mathbf{Q}}_{ij}=\mathbf{B}{\left\{\pi =i\right\}}_j1\mathrm{n}\kern0.5em \mathrm{c}-\mathbf{B}{\left\{\pi =i\right\}}_j1\mathrm{n}\kern0.5em \mathbf{B}{\left\{\pi =i\right\}}_j\\ & 1\mathrm{n}\kern0.5em p\!\left(\pi =i\left|{s}_t=j,\gamma, \mathbf{Q}\right.\right)={\mathbf{Q}}_{ij}\cdot \gamma -1 \mathrm{n} \kern0.5em {\mathrm{Z}}_{\pi}\nonumber \end{align*}

Line 2 shows how $\mathbf{Q}$ is computed for policy $i$ and state $j$. For the policy go in the G2W state and using $\mathbf{B}\left\{\mathrm{go}\right\}$ and $\mathbf{c}$ illustrated in Figure S2, this is: $0.8\cdot \ln (0.31)-0.8\cdot \ln (0.8)=-0.76$, and for no-go in this state, this is: $0.8\cdot \ln (0.003)-0.8\cdot \ln (0.8)=-4.47$ (the best policies have the minimal difference between $p\!\left({s}_T|{s}_t,\pi \right)$ and $p\!\left({s}_T|m\right)$, that is, $\mathbf{Q}$ which is negative but as close to 0 as possible). Thus, $\mathbf{Q}$ is an $i\times j$ matrix containing the KL divergence measures of the quality of $i$ allowable policies from $j$ states. The agent’s policy is then sampled from $\mathbf{Q}$ according to $p\!\left(\pi =i\right)$ and $p\!\left({s}_t=j\right)$. Note that this special (simple) case of AI reduces to a Bayes optimal state-action policy, under some prior preferences encoded by $\mathbf{c}.$

Line 3 introduces a normalizing constant ${Z}_{\pi }$ and a precision parameter $\gamma$. This encodes the confidence that desired goals can be reached, and it is inferred anew from trial to trial (Equation 14 and Figs S1, S2), based on current inferences about states, the quality of available policies and a parameter $\alpha$ quantifying the prior beliefs about confidence that participants have:

(14) \begin{equation*} {{{}^{{}^{{}^{\frown}}}}\kern-6pt{\gamma}}=\frac{\alpha }{\beta -{{{{}^{{}^{{}^{\frown}}}}\kern-6pt{\pi}}}^{\scriptsize{\rm T}}\cdot \mathbf{Q}\cdot{{{}^{{}^{{}^{\frown}}}}\kern-6pt{\rm s}}_t} \end{equation*}

where $\alpha$ is the shape parameter in a gamma-shaped prior of rate=1, that is, here is fixed to 1, so as the agent nears its goals, $\mathbf{Q}\to 0$ and thus $\gamma \to \alpha$. $\gamma$ therefore plays the role of a dynamically varying inverse temperature parameter, whose upper bound is its prior $\alpha$. We thus fractionate choice variability into two parts, one parameterized by ${c}_{\tau }$ which does not vary over time*,* and $\gamma$ which the agent must dynamically infer. For some illustrative simulations of the AI model and the role of $\alpha$, please see Figure S3, described in the Supplement.

#### Free-Energy Minimization by Agents

An agent's knowledge of how policy, states and observations interact forms a generative model over these quantities—including precision. This model is constituted by beliefs about policies—as specified by equation (13)—state transitions, the likelihood of a sequence of observations stemming from those states and prior beliefs about precision:

(15) \begin{equation*} p\!\left(\tilde{o},\tilde{s},\tilde{u},\gamma |m\right)=p\!\left(\tilde{o}|\tilde{s},m\right)p\!\left(\tilde{s}|\tilde{u},m\right)p\!\left(\tilde{u}|{s}_t,\gamma, m\right)p\!\left(\gamma |m\right) \end{equation*}

Agents invert this to infer the hidden states of the world $\tilde{s}=\left\{{s}_0,\dots, {s}_T\right\}$, to determine where each policy $\tilde{u}$ is likely to lead, and to infer the optimal precision $\gamma$ or confidence in policy selection. To do this, they use their observations $\tilde{o}$ and their model $m$ of choice-dependent probabilistic state transitions.

The variational free energy *F* of observations and beliefs under a model approximates the inconsistency of beliefs and observations: thus minimizing it maximizes the chance of achieving an agent’s goal beliefs (Friston et al. 2013). Agents can compute *F* by approximating the posterior distribution (approximating equation (15)) using a mean field assumption; namely, that subsets of variables are conditionally independent. Thus, the parameters describing belief distributions are partitioned into three common-sense subsets: statistics describing (i) beliefs about states of the world causing observations $s$, (ii) beliefs about the (future) policy $\pi$, and (iii) beliefs about precision $\gamma$. These constitute sufficient statistics $\mu =(\stackrel{\sqcup}{S}, \stackrel{\sqcup}{\pi},\stackrel{\sqcup}{\gamma})$. With each new observation, these statistics are updated iteratively in terms of the sufficient statistics of the other subsets. This approximates full Bayesian inference, and is known as variational Bayes. The update equations are shown in Figure S1*B*, alongside a putative message passing scheme that could implement these equations in the brain.

#### Model Fitting

The parameters (Fig. 2*B*) were estimated using the Hierarchical Gaussian Filter toolbox (Mathys et al. 2011), available to download from http://www.translationalneuromodeling.org/tapas/.

Parameters were appropriately transformed, so that Gaussian distributions were used at the group level. Empirical priors (Table 1) were derived by estimating the parameter distributions from the empirical data using Empirical Bayes, and iterating a small number of times until approximate convergence. Initial conditions for the empirical priors were explored over a coarse grid, to avoid local optima. The means and variances of the empirical priors are listed in Table 1. As the Pavlovian prior beliefs and $f$ are bounded by 0 and 1, they were estimated in logit space, where $\mathrm{logit}\left(x,1\right)=\ln \left(x/\left[1-x\right]\right)$. Where $a$ has a lower bound at zero, and so was estimated in log space.

**Table 1:** Prior means and standard deviations for estimation of the AI model parameters. All were estimated, although the standard deviation of ${c}_{\tau }$ was so small that it was virtually fixed

Parameter	Prior mean (native space)	Prior std dev (estimation space)
$\alpha$	1.627	0.31
$p\ \left(\mathrm{context}=\mathrm{W}\right)$	0.5163	0.14
$p\ \left({a}^{\ast }=\mathrm{go}|\mathrm{context}=\mathrm{W}\right)$	0.5211	0.21
$p\ \left({a}^{\ast }=\mathrm{no}\ \mathrm{go}|\mathrm{context}=\mathrm{AL}\right)$	0.5063	0.13
${c}_{\tau }$	5.785	0.019
$f$	0.593	0.48

We performed additional analyses to check that the model parameters could be reliably recovered from simulated datasets: these are detailed in the supplement and Figure S4*A*.

### PET Image Acquisition and Analysis

PET images were acquired using a Siemens Biograph HiRez XVI PET scanner (Siemens Healthcare). A low-dose computerized tomography scan was performed for attenuation and model-based scatter correction, followed by the injection of a single intravenous bolus of 0.020–0.029 micrograms/kg [^11^C]-(+)-PHNO. Dynamic emission data were acquired continuously for 90 min after the administration of the radiotracer. The dynamic images were then reconstructed using a filtered back-projection algorithm into 31 frames (8 × 15, 3 × 60, 5 × 120, and 15 × 300 s) with a 128 matrix, a zoom of 2.6 and a transaxial Gaussian filter of 5 mm.

PET images were analyzed using MATLAB version 2015b (Mathworks, Inc.) and MIAKAT (MIAKAT release 4.2.6, www.miakat.org; Gunn et al. 2016). An automatic pipeline was used to obtain an individual parcellation of the brain into regions of interest in MNI space, including the whole striatum and its functional subdivisions as defined by the Martinez atlas (Martinez et al. 2003). A 0–10 min [^11^C]-(+)-PHNO binding template was nonlinearly coregistered with the 0–10 min summed PET image of each participant using Statistical Parametric Mapping (SPM8 – Wellcome Trust Centre for Neuroimaging). The template was created from an internal library of [^11^C]-(+)-PHNO PET scans in healthy volunteers and normalized by individual structural MRI into standard space. A frame-by-frame registration process on a single frame of reference was used for motion correction for dynamic PET images. Individual averaged PET images were then coregistered to the 0–10 min summed PET image using rigid body coregistration. The deformation parameters from each participant’s 0–10 min [^11^C]-(+)-PHNO binding template were applied to the Martinez striatal atlas, which defines the anatomical extents of the limbic, associative and sensorimotor striatum in MNI space (Martinez et al. 2003), bringing the atlas into the individual participant's space, before it was resliced to PET resolution. Regional time activity curves (TACs) were obtained by applying the individual parcellations to the realigned dynamic images. The whole cerebellum, defined using a standard cerebellum atlas (Tziortzi et al. 2011), was used as a reference region due its low density of dopaminergic receptors (Kumakura and Cumming 2009; Egerton et al. 2010). Our outcome measure of interest was nondisplaceable binding of [^11^C]-(+)-PHNO (BP_ND_):

(16) \begin{equation*} {\mathrm{BP}}_{\mathrm{ND}}=\frac{f_{\mathrm{ND}}{B}_{\mathrm{avail}}}{K_D} \end{equation*}

where ${B}_{\mathrm{avail}}$ is the proportion of dopamine 2/3 receptors available to be bound by PHNO (i.e., the fraction of receptors not bound by endogenous synaptic dopamine), ${f}_{\mathrm{ND}}$ the free fraction of PHNO in the brain, and $1/{K}_D$ the affinity of ligand for the target. BP_ND_ for the whole and functional striatal subdivisions was obtained by kinetic modeling with a simplified reference tissue model (Lammertsma and Hume 1996; Gunn et al. 1997). Once the individual BP_ND_ maps were obtained, they were then warped back to MNI space using the inverse transformation of the initial nonlinear coregistration.

We defined the limbic striatal subdivision as our region of interest as there is a small amount of evidence from animal studies (Haluk and Floresco 2009; Ding et al. 2014) and human fMRI (Schwartenbeck et al. 2015) that it might be most important in controlling policy precision.

## Results

Healthy participants (*n* = 75) completed an orthogonalized go/no-go task (Guitart-Masip et al. 2012), which required them to learn whether to either make (go) or withhold (no-go) a response, in the context of either reward (winning money) or punishment (losing money) (Fig. 1*A*). Therefore, there were four different conditions, represented by four different abstract fractal stimuli: go to win; no-go to win; go to avoid loss; and no-go to avoid loss. On 80% of trials, feedback was provided consistent with the stimuli, while on 20% of trials, the feedback was inconsistent. About 36 trials were completed in each condition, presented in a random order. Further details are provided in the SI Appendix and elsewhere (Moutoussis et al. 2018), together with an explanation of the construction and comparison of the RL (Figs 2*A* and S1*C*) and AI (Fig. S2) models.

### Behavioral Results

The proportions of correct responses over all 75 participants in each condition (Fig. 3*A*) were consistent with previous studies (Guitart-Masip et al. 2012, 2014; Cavanagh et al. 2013). As expected, a 2 × 2 ANOVA with the factors action (go vs. no-go) and valence (reward vs. avoid loss) showed that participants were significantly more accurate on *go* than *no-go* trials (main effect of action: *F*(1,74) = 12.4, *P* < 0.001, partial *η^2^* = 0.14), with no difference between reward and punishment trials (main effect of valence: *F*(1,74) = 0.2, *P* = 0.6). There was a significant action-by-valence interaction (*F*(1,74) = 35.7, *P* < 0.0001, partial *η*^2^ = 0.32) characteristic of Pavlovian bias, with participants responding more accurately in the go to win (relative to go to avoid loss) and the no-go to avoid loss (relative to no-go to win) conditions.

We expected performance to approximately stabilize by the final 20 trials of each condition, based on previous experience (Moutoussis et al. 2018). The proportions of correct responses in these final 20 trials are shown in Figure S4*B* (performance in the final 10 trials was similar). A few participants performed substantially below chance in the more difficult conditions (go to avoid loss and no-go to win), despite performing well in the easier contexts, indicating that they learned (or inferred) incorrectly about these contexts specifically, rather than in general. Thus, we did not exclude these subjects, as we wanted to see if the models could capture the full range of individual variability.

### Computational Modeling Results

Comparing models across all 75 participants using the iBIC (Fig. 3*B*), the best was Model 7, a Rescorla–Wagner RL model that contained all parameters except decay rate $\delta$ (included parameters: reward and punishment sensitivities ${\rho}$_win_ and ${\rho}$_loss_, learning rate $\varepsilon$, irreducible noise $\xi$, go bias $b$, constant Pavlovian bias ${\pi}_c$). The AI model was fifth; it was out-performed by the four Rescorla–Wagner RL models containing a Pavlovian bias parameter. The pseudo *R*^2^ for Model 7 was 0.270 (substantially above chance); for AI, it was 0.224.

The averaged responses across all 75 participants in each condition over time are shown in Figure 3*C* (black line), along with simulated responses from the winning RL model (red line) and AI model (blue line), and their 95% confidence intervals. When simulating responses, we used each participant’s modal posterior parameter estimates 20 times (i.e., 1500 simulations in total). The Rescorla–Wagner model consistently performed better toward the start of the task, presumably because when participants are unfamiliar with the context they default to a go action (which is reflected in the go bias, $b$). Very similar results were found in the 25 subjects who also had PET imaging (Fig. S5).

**Figure 4:**
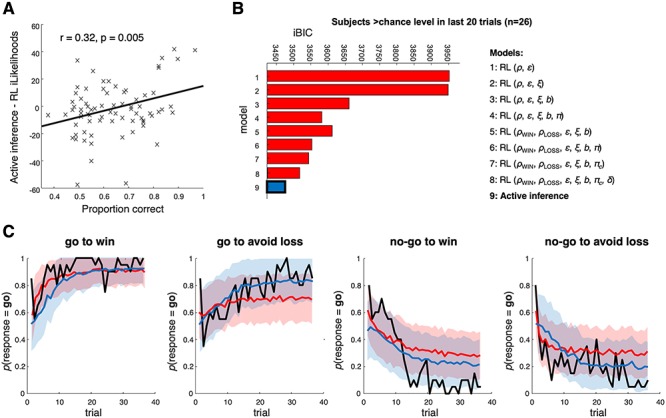
Comparing RL and AI in the participants performing above chance in the last 20 trials (*n* = 26). (*A*) The bigger the difference between AI and RL integrated likelihoods, the more correct responses participants make—that is, better performing participants will always be better fit by AI, whatever performance threshold is chosen. (*B*) Model comparison in only the participants who were >50% correct (i.e., above chance) in the final 20 trials in each condition (*n* = 26)—here, the AI model wins by a reasonable margin (iBIC of 42). Model 8 is in second place. (*C*) These plots compare the actual probability of participants’ go responses (averaged across participants who were >50% correct in the last 20 trials (*n* = 26)) with model predictions (from Model 8, the winning RL model in this group, and Model 9, AI) in each condition, in the same format as the previous figure. Each model used each participant’s estimated parameters 58 times, that is 1508 times in total. 95% confidence intervals for the means of the simulations (shaded areas) were derived from the distributions of means of 100 samples of *n* = 26 participants. RL tends to fit the start of the sequences better, but AI better fits the rest of the sequences, because (i) the Pavlovian priors get overridden by the data in the best participants, (ii) participants can become more deterministic as they accumulate knowledge about the task.

Conversely, the behavior of participants who accurately inferred—or learned—the task was better explained by AI. Across participants, there was a significant positive association between the difference in model evidence between AI and RL Model 7 and the proportion of correct choices (*r* = 0.32, *P* = 0.005; Fig. 4*A*). Consistent with this, the 26 participants who achieved >50% correct responses in every condition (i.e., above chance in all four contexts) across the final 20 trials clearly favored AI over the next best RL model (Fig. 4*B*). NB all 36 trials were used for model inversion in these subjects, not just the final 20. Similar results obtained if other thresholds were chosen, for example, >60% or >70% correct responses, and/or using the last 10 trials.) Interestingly, the best-fitting RL model in these better-performing subjects was Model 8, which included a forgetting process. The pseudo *R*^2^ for the AI model in these 26 participants was 0.334 (for Model 8 it was 0.326).

We also found that a purely data-driven method also picked out subjects best fit by AI. We grouped participants using *k*-means clustering according to accuracy on the last 20 trials in each condition. This produced four clusters of participants, with AI best fitting the two largest clusters (Fig. S6): a group of ‘high performers’ (cluster 4: *n* = 31); and a group who were more accurate in go than no-go contexts (*n* = 23).

Thus, AI better accounted for behavior relative to RL as performance improved. Figure 4*C* shows the averaged responses of the 26 most accurate participants and simulated data in the same format as Figure 3*C*. Although the Rescorla–Wagner model fits the initial actions of each context better, the AI model quickly improves on it in three out of four conditions. Note that in Figure 4*C*, we are comparing AI with RL Model 8, but all subsequent comparisons are with Model 7, the best RL model overall.

To further understand the difference between the models, we derived summary behavioral measures of deterministic responding and of the number of trials before participants settled on one response—termed ‘normalized switches’ and ‘trials to decision point’ respectively (see Methods). There were strong correlations between the evidence for (i.e., integrated likelihood of) AI and all measures (normalized switches: *r* = 0.88, *P* = 10^−25^; trials to decision: *r* = –0.71, *P* = 10^-12^; Fig. S7*A*). The correlations between these measures and RL model likelihood (normalized switches: *r* = 0.52, *P* = 10^-6^; trials to decision: *r* = –0.36, *P* = 0.002; Fig. S7*A*) were both significantly weaker (Steiger’s *Z*, *P* = 10^-6^ and *P* = 0.00004, respectively).

Mediation analyses revealed that making consistent responses and deciding early was favored more by AI than RL, irrespective of whether these responses were actually correct (Fig. S7*B*). The degree to which a subject’s model evidence favored AI over RL Model 7 did not significantly relate to their digit span (*r* = 0.25, *P* = 0.13) or IQ estimated from the WTAR (*r* = 0.14, *P* = 0.4), however.

The importance of consistency for AI model fits can also be observed in individual participants’ action sequences: even when performance is matched across participants (Fig. S8*A*), AI accounts for participants who are consistent (even if they are wrong about one or two contexts—for example, participants 45 and 65, Fig. S8*B*), whereas the RL model better explains the behavior of people who respond more inconsistently (e.g., participants 57 and 62, Fig. S8*B*).

Given that neither AI nor RL models are perfect, and each may capture distinct aspects of behavior, we performed a factor analysis (see Supplementary Methods) on their combined parameters to see if a ‘choice stochasticity’ factor emerged, in order to assess its relationship to D_2/3_R availability. The AI prior over policy precision, $\alpha$,$alpha$ and the RL reward sensitivity parameter, ${\rho}$_win_, were strongly associated (*r* = 0.47, *P* < 0.00076, i.e., Bonferroni corrected for 66 comparisons), and these parameters loaded strongly on the first factor, along with the RL irreducible noise parameter, $\xi$ (Fig. S9*A* and *B*). The Pavlovian parameters in both models loaded on the second factor, and RL loss sensitivity, ${\rho}$_loss_, strongly loaded on the third factor.

Finally, the AI forgetting parameter, $f$, correlated with both IQ (*r* = –0.37, *P* = 0.0015) and Digit Span (*r* = –0.36, *P* = 0.0021): both significant after Bonferroni correction for 12 comparisons (*P* < 0.0042). The correlation of $f$ with IQ was not driven by the best-performing participants; in the participants performing at or below chance in the last 20 trials (*n* = 49), whose behavior was fit best by RL, the correlation was *r* = –0.30, *P* = 0.059. The AI prior on policy precision parameter, $\alpha$, did not correlate with either measure (*r* = 0.09 and 0.21, respectively; both *P* > 0.05). In RL Model 7, only go bias, $b$, correlated with Digit Span (*r* = 0.28, *P* = 0.020)—but not with IQ (*r* = 0.15, *P* = 0.2)—and this did not survive Bonferroni correction.

### PET Results

Τhe behavioral performance of the 25 participants in the PET study was similar to the entire sample: the same 2 × 2 ANOVA revealed that participants showed a significant action-by-valence interaction (*F*(1,24) = 17.0, *P* < 0.0001). Again, there was no main effect of valence (*F*(1,24) = 0.2, *P* = 0.7), but in this subset the main effect of action did not reach statistical significance (*F*(1,24) = 2.7, *P* = 0.11). RL Model 6 narrowly beat RL Model 7 (iBICs of 6073 and 6086, respectively), with the AI model in fifth position, behind all the models with Pavlovian biases. Averaged responses in each of the four contexts—and simulated data from each model—also resembled those in the entire group (Fig. S5).

**Figure 5:**
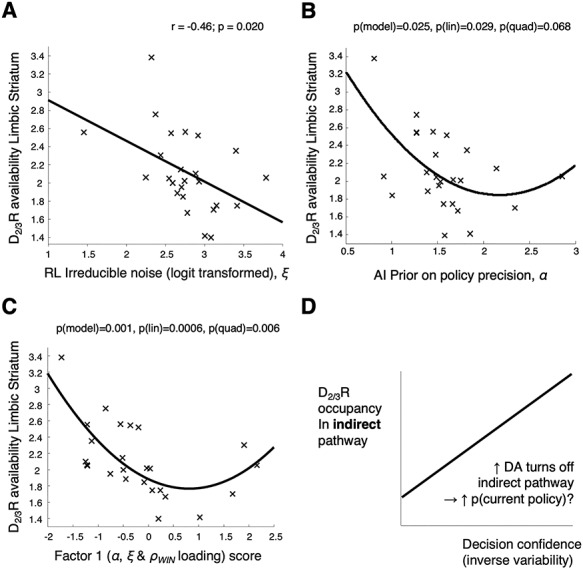
Relationships between D_2/3_R availability and RL and AI parameters governing response stochasticity in participants who completed PET (*n* = 25). (*A*) There is a linear relationship between irreducible decision noise $\xi$ and limbic striatal D_2/3_R availability (measured as [^11^C]-(+)-PHNO BP_ND_) in the participants who completed a PET scan (*r* = –0.46, 95% CI [–0.72 –0.08], *P* = 0.020). Here, $\xi$ was logit transformed as it was not normally distributed: $\mathrm{logit}\left(\xi \right)=\ln \left(\xi /\left[1-\xi \right]\right)$. Without the transform, the correlation remained significant (*r* = –0.45, 95% CI [–0.72 –0.07], *P* = 0.023). (*B*) There is a significant linear relationship between the prior on policy precision $\alpha$ and D_2/3_R availability, *β*_linear_ = –2.1, SE = 0.91, *t*_22_ = –2.33, *P* =0.029; there is also evidence of a quadratic relationship but it is not significant, *β*_quad_ = 0.49, SE = 0.26, *t*_22_ = 1.92, *P* = 0.068. (*C*) This plot shows the relationships between D_2/3_R availability and the first factor in the factor analysis of the AI and RL parameters (detailed in Fig. S9). Three parameters governing response stochasticity loaded on this factor (loadings in brackets) – $\alpha$ (0.69), ${\rho}$_win_ (0.88), and $\xi$ (0.30)—and this factor has robust linear (*β*_linear_ = –0.29, SE = 0.07, *t*_22_ = –3.97, *P* = 0.0006) and quadratic (*β*_quad_ = 1.8, SE = 0.06, *t*_22_ = 3.06, *P* =0.006) relationships with D_2/3_R availability. (*D*) We interpret the linear relationships in plots A–C as being due to greater tonic D_2_R occupancy suppressing the indirect pathway more, hence making participants’ choices more deterministic.

Considering the RL model, we detected the expected significant negative linear relationship between limbic striatal D_2/3_R availability and $\xi$ (*r* = –0.46, *P* = 0.020) but no quadratic relationship (Fig. 5*A*). Contrary to our predictions, there were no significant relationships between limbic striatal D_2/3_R availability and ${\rho}$_win_ or ${\rho}$_loss_.

From the AI model, we detected the expected significant negative linear relationship between limbic striatal D_2/3_R availability and $\alpha$ (*P* = 0.029), while the quadratic term narrowly missed significance (*P* = 0.068; overall model *F*(2,22) = 4.4, *P* = 0.025, *R*^2^(adj) = 0.22; Fig. 5*B*). The linear relationship was significant without the quadratic term (*r* = –0.41, *P* = 0.043).

Finally, we found that the ‘choice stochasticity’ factor derived from the factor analysis across parameters from the AI and RL models – on which $\alpha$, ${\rho}$_win_ and $\xi$ all loaded (with scores of 0.69, 0.88 and 0.30 respectively; Fig. S9*B*) – had highly significant linear (*P* = 0.0006) and quadratic (*P* = 0.006) relationships with limbic striatal D_2/3_R availability (overall *F*(2,22) = 9.5, *P* = 0.001, *R*^2^(adj) = 0.41; Fig. 5*C*). Again, the linear relationship was significant without the quadratic term (*r* = –0.48, *P* = 0.014). Parameter recovery analysis (using simulated data – see the supplement) showed these parameters could be reliably recovered, with correlations between simulating and estimated parameters of *r* = 0.81 for $\alpha$, *r* = 0.74 for ${\rho}$_win_, and *r* = 0.51 for $\xi$ (Fig. S4*A*). None of the other factors had significant relationships with limbic striatal D_2/3_R availability.

In exploratory analyses, we found that the ‘choice stochasticity’ factor also had significant relationships with D_2/3_R availability in the associative striatum and across the striatum as a whole, but not within the sensorimotor striatum (detailed in the Supplement). We also found a relationship we had not hypothesized between RL learning rate $\varepsilon$ and limbic striatal D_2/3_R availability (Fig. S9*C*), but this was not significant after multiple comparisons correction. It is detailed in the Supplement. No other parameters had a relationship with limbic striatal D_2/3_R availability.

## Discussion

Consistent with our hypothesis, using both RL and AI models of a challenging go/no-go task we found clear negative relationships between D_2/3_R availability in the limbic striatum and choice stochasticity parameters. These were the RL irreducible noise parameter ($\xi$) and the AI prior precision over policies parameter ($\alpha$). Notably, a ‘choice stochasticity’ factor (derived from a factor analysis of parameters across the models) had highly significant linear and quadratic relationships with D_2/3_R availability. This is consistent with the hypothesis that D_2/3_R occupancy in the indirect pathway decreases choice variability (although D_2/3_R density also contributes to D_2/3_R availability). In model comparison, the Rescorla–Wagner RL model won overall, but participants’ performance correlated with the degree to which AI outperformed Rescorla–Wagner in modeling their behavior. AI especially favored participants who responded more consistently (i.e., less stochastically) and who decided more quickly on the appropriate action for the context. Finally, we also observed positive linear relationships between D_2/3_R availability in the limbic striatum and an AI ‘Pavlovian’ prior belief parameter ($p\!\left({a}^{\ast }=\mathrm{go}|\mathrm{context}=\mathrm{W}\right)$), as well as the RL learning rate ($\varepsilon$).

### Choice Stochasticity Parameters Negatively Relate to Limbic Striatal D_2/3_R Availability

The negative linear relationship between the ‘choice stochasticity’ factor (on which policy precision $\alpha$, reward sensitivity ${\rho}$_win_ and irreducible noise $\xi$ loaded) and D_2/3_R availability in the limbic striatum (Fig. 5*C*) is an important finding. We predicted this relationship a priori, because in the AI framework, dopamine is thought to encode the precision over policies ($\gamma$), whose upper bound is $\alpha$. We therefore hypothesized that $\alpha$ would be encoded by tonic striatal dopaminergic activity, which impacts D_2/3_R availability. This is consistent with the hypothesis that the indirect pathway promotes switching among policies (see below), since D_2/3_R activity inhibits this pathway, making actions more deterministic (Fig. 5*D*). Therefore, if D_2/3_R availability is at least in part an inverse measure of this tonic activity, there should be a negative relationship between choice stochasticity and D_2/3_R availability. Note, however, that a dopamine-depletion PET study would be required to conclusively establish that the negative relationship between D_2/3_R availability and choice stochasticity is due to tonic activity at these receptors, rather than receptor density (see below).

It is not surprising that the reward sensitivity parameter, ${\rho}$_win_, loaded strongly on the choice stochasticity factor, as ${\rho}$_win_ is equivalent to a decision inverse temperature parameter in the win domain. Consistent with this, it also correlated strongly with $\alpha$ (*r* = 0.47). Although ${\rho}$_win_’s relationship with D_2/3_R availability was nonlinear and not captured by linear or quadratic regression, there was a linear relationship between irreducible noise ($\xi$) and D_2/3_R availability (Fig. 5*A*).

There was also a quadratic relationship between the choice stochasticity factor and D_2/3_R availability, and between $\alpha$ and D_2/3_R availability (although the latter narrowly missed statistical significance – Fig. 5*B*). The quadratic relationships are not important for our analyses, but they could arise from individual differences in either receptor density (as BP_ND_ is a function of both tonic dopamine activity and the total density of D_2/3_Rs) or presynaptic D_2/3_R autoreceptors (Fig. 1*B*) that decrease dopamine release (Ford 2014). Previous studies have observed similar nonlinear relationships between striatal D_2/3_R availability and the ability to change a policy following negative outcomes (Groman et al. 2011; Cox et al. 2015).

### Model Comparison Favors RL Over AI Overall, But AI in Accurate Participants

This is the first study to directly compare the performance of RL and AI models using empirical data (also see FitzGerald et al. 2015). This was not intended to be a comprehensive comparison (e.g., model-based or ‘planning as inference’ RL models could be constructed), but one focused on the specific issues of accounting for response variability and biases. Nonetheless, instructive conclusions can be drawn. First, while Rescorla–Wagner RL was favored over all participants, a higher likelihood of AI relative to RL was associated with more accurate performance: in participants performing above chance in the last 20 trials of each condition, the AI model was favored. Although only a third of subjects achieved this performance in every condition, our sample performed similarly overall to previous samples, given earlier versions employed more trials and hence got closer to a performance asymptote.

Additionally, independent of accuracy, greater evidence for the AI model was associated with more consistent responding (i.e., less switching), and a lower number of trials to reach a ‘decision point’ (Fig. S7*B*). This pattern is likely to arise because the Bayesian AI model can (i) incorporate prior knowledge about the task structure, (ii) dynamically adjust both the rate at which it updates its beliefs and the stochasticity with which it chooses its actions, according to its uncertainty, and (iii) ‘overwrite’ its Pavlovian prior beliefs about the prevalence of contexts. In contrast, the Rescorla–Wagner RL model uses fixed learning and decay rates and decision noise and reward/punishment sensitivity parameters to fit the entire sequence of trials, and its decision biases (${\pi}_c,b$) are expressed during action selection, and hence are hard to overcome. That said, the AI model might benefit from a similar ‘go bias,’ which would likely improve its fitting of the initial trials, when subjects prefer to explore using ‘go.’ Last, it is possible that some of AI’s superior performance in the more accurate subjects is due to its ‘forgetting’ process, given that incorporating forgetting improved RL Model 8s fitting of these subjects. Nevertheless, AI still outperformed Model 8, probably due to the Bayesian factors highlighted above.

The degree to which subjects were favored by AI or Rescorla–Wagner RL models did not relate to their age, digit span or estimated IQ. Nevertheless, the existence of subgroups with distinctive patterns of performance and model fits (Fig. S6), indicates some subgroups might be separable using other tasks or measures: for example, ‘sign-tracking’ and ‘goal-tracking’ in Pavlovian conditioning paradigms (Flagel et al. 2007). These groups (found in rodents and humans) are best described by model-free and model-based RL respectively (Schad et al. 2019).

We emphasize that we contrasted prototypical AI with successful Rescorla–Wagner RL models of this task, rather than adding further parameters to either, or drilling down to their irreducible differences (Gershman 2019). Model-based RL incorporates explicit Bayesian updating of beliefs about states (Dayan et al. 2000; Gershman 2017) and is thus closer to AI. With respect to specific parameters, some versions of AI have incorporated an irreducible noise parameter (Mirza et al. 2018), while numerous RL models have incorporated forgetting (Niv et al. 2015; Kato and Morita 2016; de Boer et al. 2017), and a decision temperature parameter that decreases during learning (Crites and Barto 1995). Other RL-type models have incorporated approximate Bayesian features ‘by hand’, for example, learning rates informed by uncertainty (Pearce and Hall 1980), or aspects of belief-based learning (Camerer and Ho 1999).

### Other Work Supports Striatal D_2/3_Rs Encoding the Precision of Action Selection

Only one other neuroimaging study has looked at policy precision encoding (using AI) in the brain (Schwartenbeck et al. 2015): in a gambling task, policy precision updates correlated with fMRI activity in the substantia nigra (SN) and ventral tegmental area. Importantly BOLD activity in these dopaminergic regions may itself be related to midbrain D_2/3_R availability (Nour et al. 2018). This result survived the inclusion of expected value and current reward in the model. As decision stochasticity (i.e., softmax temperature) is usually a fixed parameter in RL models, it cannot be used as a time-varying fMRI regressor, and so its relationship to the brain is rarely explored using neuroimaging.

Pharmacological studies have provided evidence of such a relationship, however. Eisenegger et al. (2014) showed—using a standard probabilistic bandit task and a Q-learning model—that a moderate dose of a D_2_R antagonist (sufficient to block postsynaptic striatal D_2_Rs; Mehta et al. 2008) increased decision temperature for gains (but not losses), which is consistent with the relationship we observed between D_2/3_R availability and Factor 1 (containing ${\rho}$_win_ but not ${\rho}$_loss_). In participants with high serum levels of the drug, the increase in decision temperature was particularly marked. T/T homozygotes for the DARPP-32 gene, who are thought to have increased striatal dopamine, show reduced random exploration in a bandit task (Gershman and Tzovaras 2018). This is similar to findings in macaques (Lee et al. 2015) in which specific D_1_R and D_2_R antagonists were injected directly into the dorsal striatum: only the D_2_R antagonist increased decision temperature. D_2_R knockout mice also have increased decision temperature (Kwak et al. 2014). Finally, sustained plateaus in dopaminergic firing in SN (preferentially detected by striatal D_2_Rs) correlate with decision variability (not average reward rate), and chemogenetically-induced increases in this firing promote exploitative responding (Koralek and Costa 2019).

Taken together, the above studies are consistent with the notion that transmission at striatal D_2_R receptors increases the precision of—that is confidence in—action selection. Of course, this can also be interpreted as increasing reward sensitivity (Averbeck and Costa 2017): for example, disrupting D_1_R-expressing neuron function in mice during a probabilistic RL task increases decision temperature (Cieślak et al. 2018), but this could also be seen as reducing the amplitude of reward prediction errors (as reward sensitivity does). The ‘policy precision’ account, however, is a better explanation of the finding that a D_2_R antagonist specifically disrupts the trial-by-trial relationship between high-level uncertainty (phasic volatility) estimates in a motor sequence task and reaction time (Marshall et al. 2016).

However, it should be acknowledged that some studies have not found a relationship between D_2_Rs and choice stochasticity (Wunderlich et al. 2012; Costa et al. 2015), and a few findings imply the opposite of our results: D_2_R and D_3_R agonists caused increased response variability in rats (Pesek-Cotton et al. 2011; Stopper et al. 2013), and ‘hyperdopaminergic’ (dopamine transporter knockdown) mice had increased ‘stay’ decision temperatures (Beeler et al. 2010) (but see Cagniard et al. 2006). D_2_R antagonism can also block punishment-induced increases in movement variability (Galea et al. 2013). Some complicating factors in these experiments are: (i) at lower doses, D_2/3_R drugs act preferentially on autoreceptors (Ford 2014), thus opposing their postsynaptic actions, and (ii) very high (especially nonphysiological) concentrations of dopamine make behavior more disordered, not less, resulting in an inverted-U relationship between dopamine and choice consistency (Cools and D’Esposito 2011).

Given these complicating factors, it is helpful to view dopamine’s function from a computational perspective. Whether dopamine neuron firing increases in response to reward prediction errors (as in RL) or confidence in one’s policy (as in AI), in both cases it makes sense for physiological concentrations of dopamine to reduce the stochasticity of actions, as modeling work implies (Humphries et al. 2012; Fiore et al. 2016), by facilitating the current policy and inhibiting competing ones (Haluk and Floresco 2009; Cui et al. 2013; Ott et al. 2014; Howard et al. 2017). This is analogous to its hypothesized role in controlling the signal/noise in cortical representations of states (Redish et al. 2007; Durstewitz and Seamans 2008). Indeed, given Findling et al.'s (2019) striking finding that >50% of choice variability in a volatile bandit task is attributable to noise in the RL learning process (rather than exploration or decision noise), it may be that catecholamines like dopamine and noradrenaline can regulate the variance in learning/inference itself.

One might expect that D_2/3_R availability in the associative striatum would relate to policy precision, rather than the limbic part (as we observed). Both parts have been implicated in the control of decision temperature; however, injecting a D_2_R antagonist into dorsal striatum increases decision temperature (Lee et al. 2015), but disrupting transmission (using an NMDA receptor antagonist) in ventral—but not dorsal—striatum impairs response switching in rats (Ding et al. 2014). Likewise, injecting a D_2_R agonist into ventral striatum in rats impairs behavioral flexibility (Haluk and Floresco 2009).

### Interpreting the [^11^C]-(+)-PHNO Signal

Given that [^11^C]-(+)-PHNO binds to both D_2_ and D_3_ receptors, one might question whether the correlations between [^11^C]-(+)-PHNO BP_ND_ and parameters were driven by D_2_Rs or D_3_Rs, and whether this affects the interpretation of the results. D_2_Rs and D_3_Rs are related to – and have similar inhibitory effects on—their target neurons. Although [^11^C]-(+)-PHNO has much higher affinity for D_3_Rs than D_2_Rs (Rabiner et al. 2009), the scarcity of the former in the striatum means that only around 20% of the ventral striatal and almost none of the dorsal striatal [^11^C]-(+)-PHNO signal relates to D_3_Rs (Tziortzi et al. 2011). While the indirect pathway is characterized exclusively by D_2_R expression, both D_2_Rs and D_3_Rs serve as autoreceptors (Beaulieu and Gainetdinov 2011), although the latter probably have a more minor role (Ford 2014).

We have interpreted the relationships we detected between [^11^C]-(+)-PHNO BP_ND_ and computational parameters as being driven primarily by tonic D_2/3_R occupancy by dopamine, rather than D_2/3_R density, because it seems most likely that activity at those D_2/3_Rs—rather than their density—mediates brain computations. A PET study with and without dopamine-depletion would be required to conclusively demonstrate this, however. Also, as an agonist tracer, [^11^C]-(+)-PHNO is more sensitive to synaptic dopamine levels than antagonist tracers such as [^11^C]raclopride (Shotbolt et al. 2012). Importantly, the correlation between [^11^C]-(+)-PHNO BP_ND_ and tonic D_2/3_R occupancy (estimated using [^11^C]-(+)-PHNO BP_ND_ before and after dopamine depletion with alpha-methyl-para-tyrosine (α-MPT)) in dorsal and ventral striatum is estimated at around *r* = –0.7 or greater (Caravaggio et al. 2016). This implies that at least 50–60% of the variance (probably more, as dopamine depletion is never total) in striatal [^11^C]-(+)-PHNO BP_ND_ is accounted for by tonic D_2/3_R occupancy.

### Limitations

The main limitation of this study is that PET is correlative and [^11^C]-(+)-PHNO BP_ND_ in particular does not only reflect synaptic dopamine levels, but also receptor density. Nevertheless, PET is the only technique available to study dopamine function in vivo in healthy humans. Also, given its cost, we could not scan all the participants who performed the task: we therefore could not examine relationships between [^11^C]-(+)-PHNO BP_ND_ and AI parameters in the best-performing participants, which may be more robust. There was also a delay of up to 15 days between the behavioral testing and the PET scan: this could potentially introduce some variance. The test–retest reliability of [^11^C]raclopride binding in the striatum is >0.8 over a 5-week-period (Alakurtti et al. 2015), but a similar timeframe has not been studied using [^11^C]-(+)-PHNO.

Furthermore, the nature of PET scanning means that we were only able to obtain single measures of dopaminergic function for each participant, which we have interpreted as reflecting tonic dopamine levels. This can be contrasted with temporally resolved measures of dopaminergic responses (e.g., voltammetry or dynamic displacement studies) that would allow a more fine-grained analysis of induced dopaminergic responses and their computational roles.

The rationality of the Bayesian models that we used may need further bounding (i.e., beyond ‘forgetting’) to faithfully describe brain processes. Also, most participants may not begin the task with perfect representations of the transition probabilities, no matter how well-instructed they are, but become more accurate during the task. This could be modeled in AI by an increasing precision of beliefs about the transition probabilities over time (FitzGerald et al. 2015). Likewise, one could add Bayesian inference of states to the RL model.

## Conclusion

We investigated how striatal D_2/3_Rs may control the variability of action selection, using [^11^C]-(+)-PHNO PET imaging and computational models of behavior in a go/no-go task: namely, AI and Rescorla-Wagner RL. AI contains a precision over policies ($\gamma$) that is similar in function to decision inverse temperature (or outcome sensitivity) in RL models, but is dynamically updated during the task, as participants become more confident. In the 25 participants undergoing PET imaging, we found a negative relationship between participants’ priors on $\gamma$ and D_2/3_R availability in limbic striatum, as predicted by AI process theories. We also observed a negative linear relationship between decision noise in RL and D_2/3_R availability in limbic striatum; and policy precision, decision noise, and reward sensitivity all loaded on a common factor that negatively correlated with this D_2/3_R availability. These findings are consistent with the occupancy of inhibitory D_2/3_Rs in the striatal indirect pathway reducing the stochasticity of action selection (or, in AI terms, increases policy precision).

## Author Contributions

RAA, MM, KJF, ODH, and JPR conceived the experiment; RAA, TD, MN, DL, and BI acquired the data; RAA, MM, TD, MN, DL, BI, CM, MV, LdB, and MG analyzed the data; RAA wrote the paper with contributions from all other authors.

## Funding

RAA is supported by the Academy of Medical Sciences (AMS-SGCL13-Adams), the National Institute of Health Research (CL-2013-18-003), and the NIHR UCLH Biomedical Research Centre. MM is staff in the ‘Neuroscience in Psychiatry Network’ project, granted by the Wellcome Strategic Award (ref 095844/7/11/Z) to Raymond Dolan and others; The Max Planck–UCL Centre for Computational Psychiatry and Ageing is a joint initiative of the Max Planck Society and UCL. MM also receives support from the NIHR UCLH Biomedical Research Centre. MN and TD are supported by the National institute for Health Research. TD is supported by an EU-FP7 MC6 ITN IN-SENS grant (grant number 607616). MGM and LdB were supported a research grant from the Swedish Research Council, VR521-2013-2589 (MGM). KJF is funded by the Wellcome Trust (Ref: 088130/Z/09/Z). This study was funded by a NIHR UCLH Biomedical Research Centre pump priming award to JPR (BRC252/NS/JR/101410), and Medical Research Council-UK (no. MC-A656-5QD30) and Wellcome Trust (no. 094849/Z/10/Z) grants to ODH and the National Institute for Health Research Biomedical Research Centre at South London and Maudsley NHS Foundation Trust and King’s College London. The funders had no role in study design, data collection and analysis, decision to publish, or preparation of the manuscript.

## Notes

We are grateful to Prof. Ray Dolan for the use of task code from the Neuroscience in Psychiatry Network project. We thank Peter Dayan for useful discussions. We also thank the MR and PET technologists and radiographers at Imanova Centre for Imaging Sciences (Invicro Ltd), London, and also Dr Graham Searle and Dr Christopher Coello for help with the PET analysis. *Conflict of Interest*: ODH has received investigator-initiated research funding from and/or participated in advisory/speaker meetings organized by Astra-Zeneca, Autifony, BMS, Eli Lilly, Heptares, Jansenn, Lundbeck, Lyden-Delta, Otsuka, Servier, Sunovion, Rand, and Roche. Neither O.H. nor his family have been employed by or have holdings/a financial stake in any biomedical company. The other authors declare no competing financial interests.

## Significance Statement

All computational models of decision-making contain a mechanism that controls how randomly or deterministically actions are chosen, given the quality of the options available. In RL models, the most ubiquitous form of decision-making model, this ‘variability control’ mechanism is encoded in decision ‘noise’ and/or ‘outcome sensitivity’ parameters. In AI, a Bayesian decision-making model, a ‘policy precision’ variable plays a very similar role. The biological basis of this control over action selection variability is unclear, however. Here, we use PET imaging of striatal dopamine 2/3 receptors and computational modeling of healthy human subjects’ behavior in a go/no-go task to show that dopamine 2/3 receptor availability strongly relates to decision variability, whichever model of behavior is used.

## Supplementary Material

FigureS1_bhz327

FigureS2_bhz327

FigureS3_bhz327

FigureS4_bhz327

FigureS5_bhz327

FigureS6_bhz327

FigureS7_bhz327

FigureS8_bhz327

FigureS9_bhz327

Supplement_v4_bhz327
