# Supplementary figures and images for: Variability in Action Selection Relates to Striatal Dopamine 2/3 Receptor Availability in Humans: A PET Neuroimaging Study Using Reinforcement Learning and Active Inference Models
Source: Cereb Cortex. 2020 Feb 21;30(6):3573–89. doi: 10.1093/cercor/bhz327 (PMC7233027; doi:10.1093/cercor/bhz327)

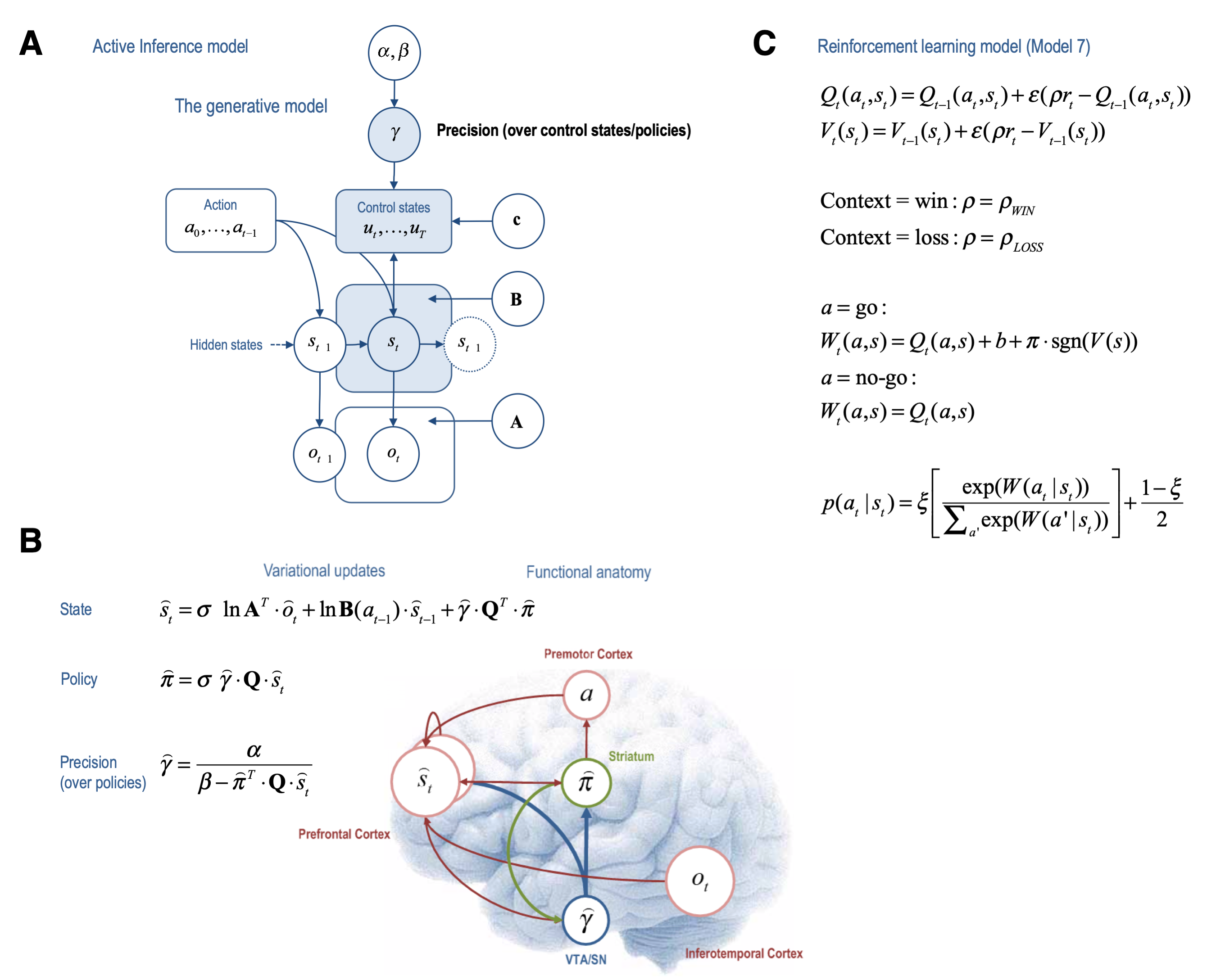

Supplement: FigureS1_bhz327 [file figures1_bhz327.png]

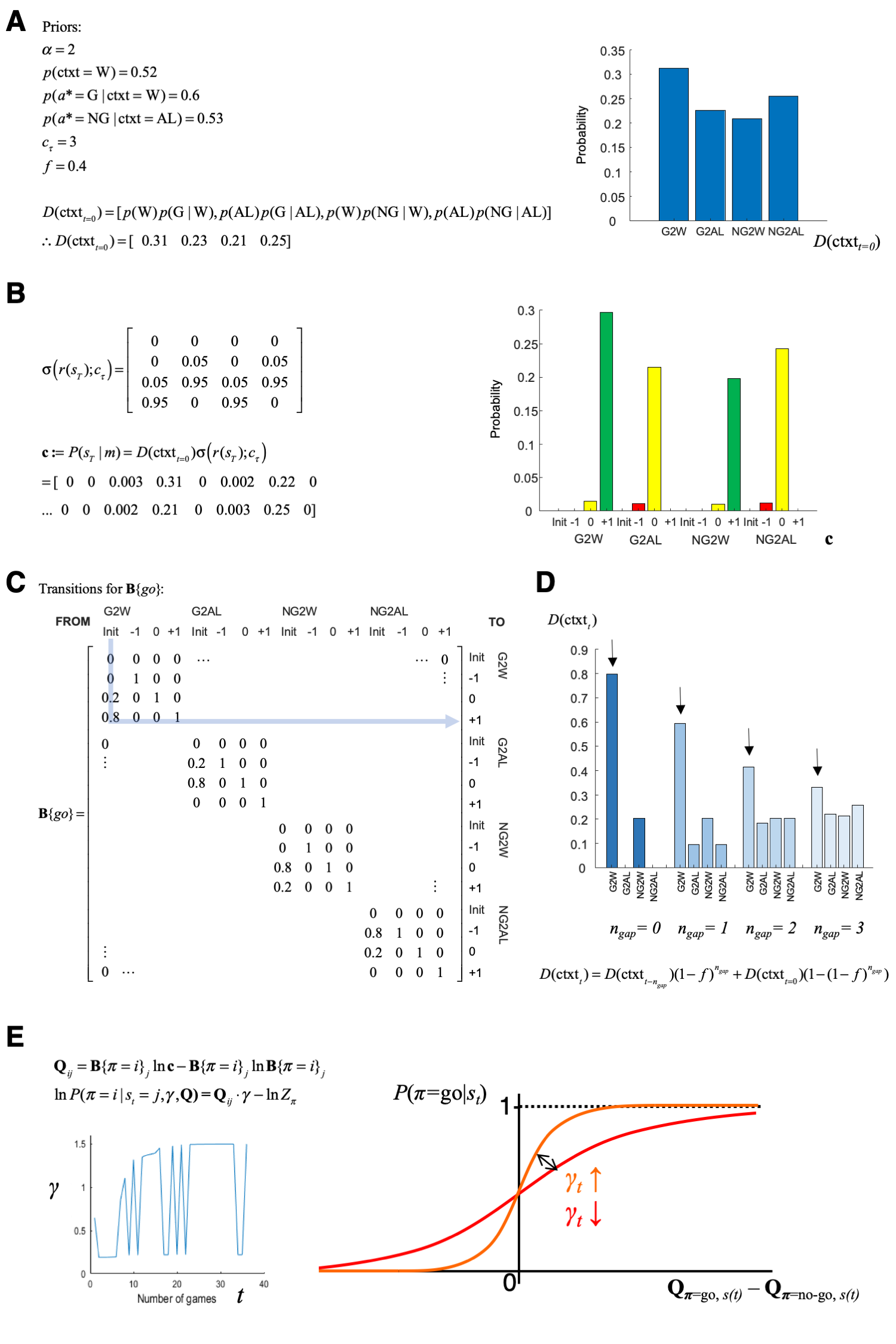

Supplement: FigureS2_bhz327 [file figures2_bhz327.png]

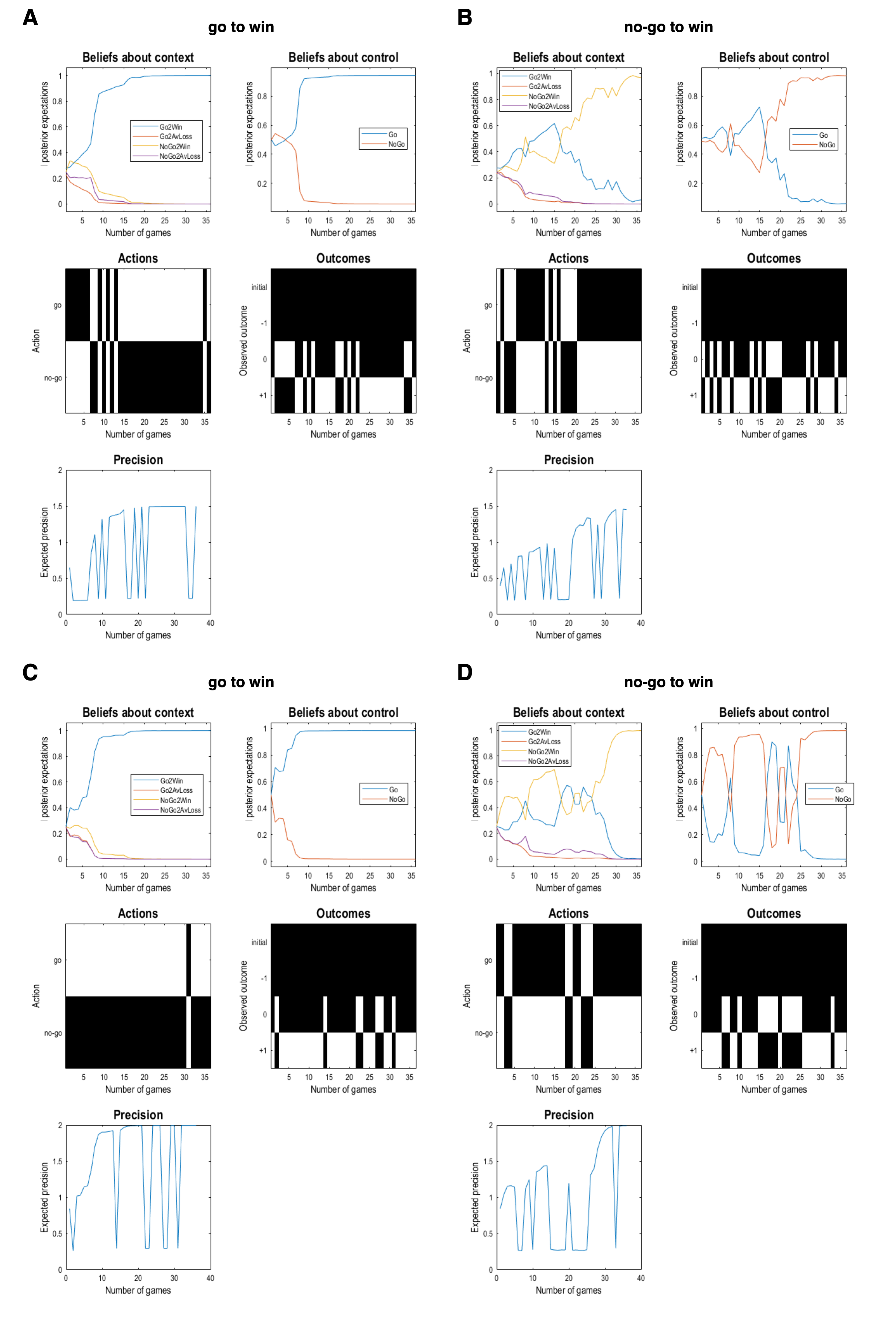

Supplement: FigureS3_bhz327 [file figures3_bhz327.png]

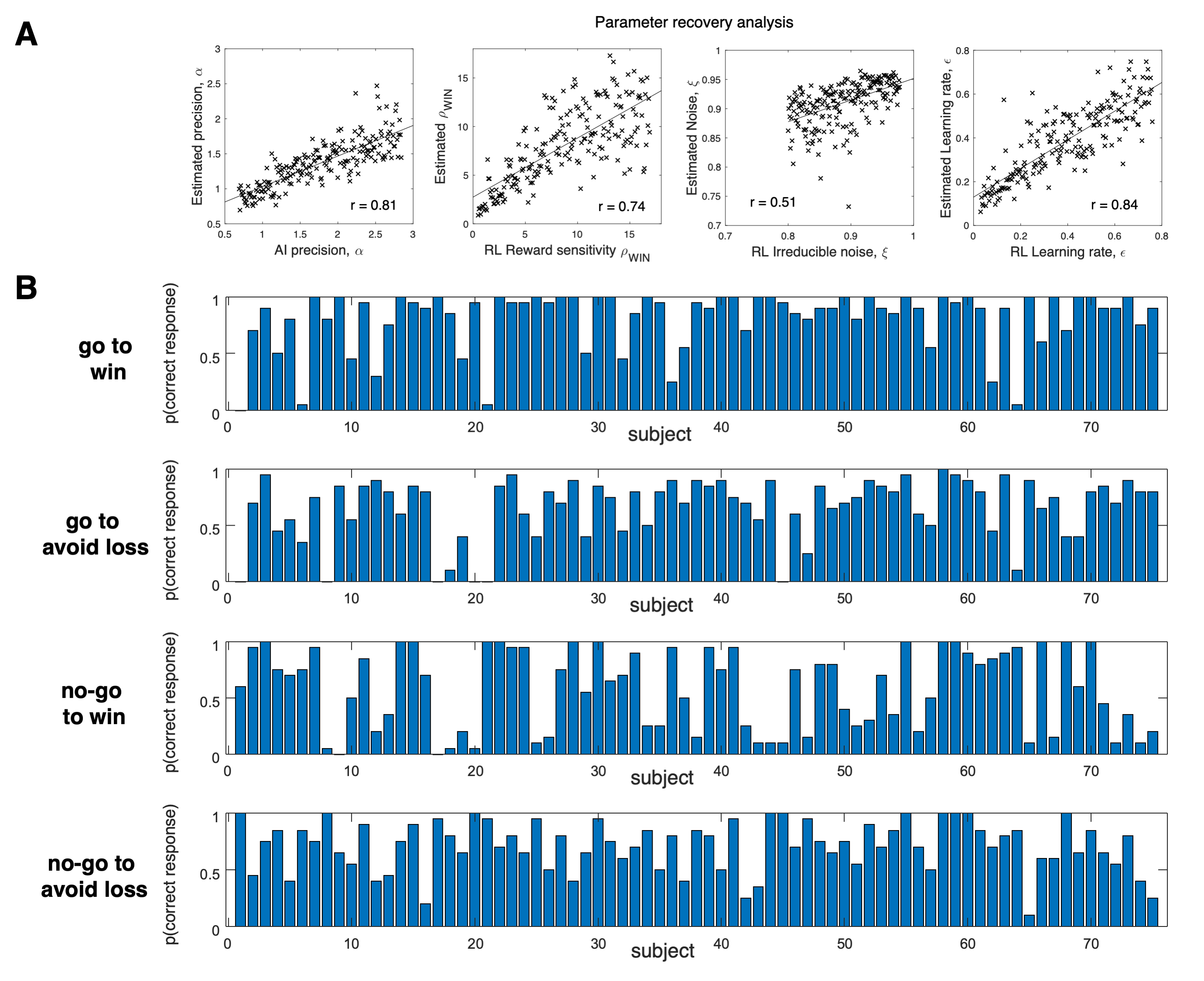

Supplement: FigureS4_bhz327 [file figures4_bhz327.png]

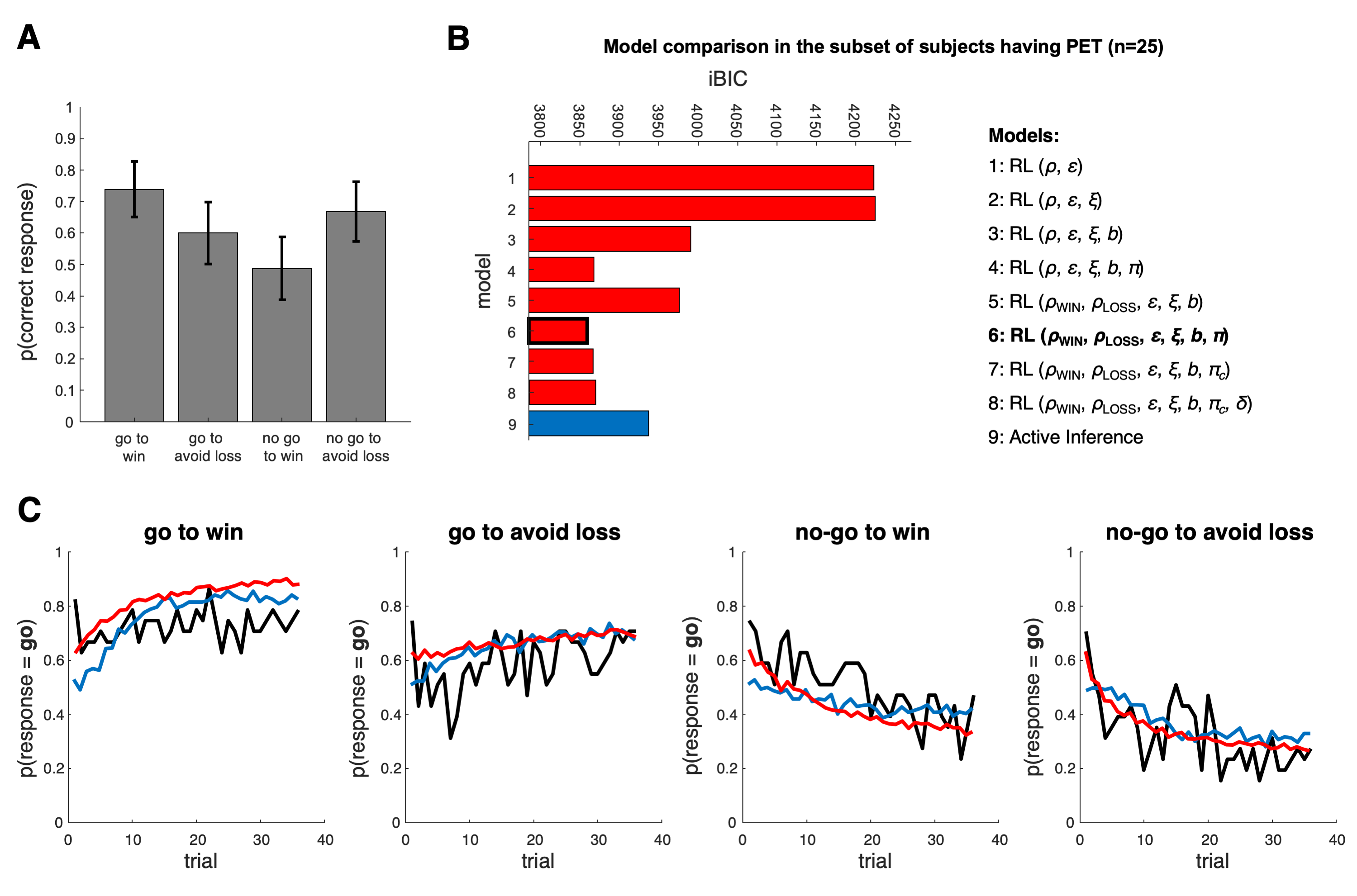

Supplement: FigureS5_bhz327 [file figures5_bhz327.png]

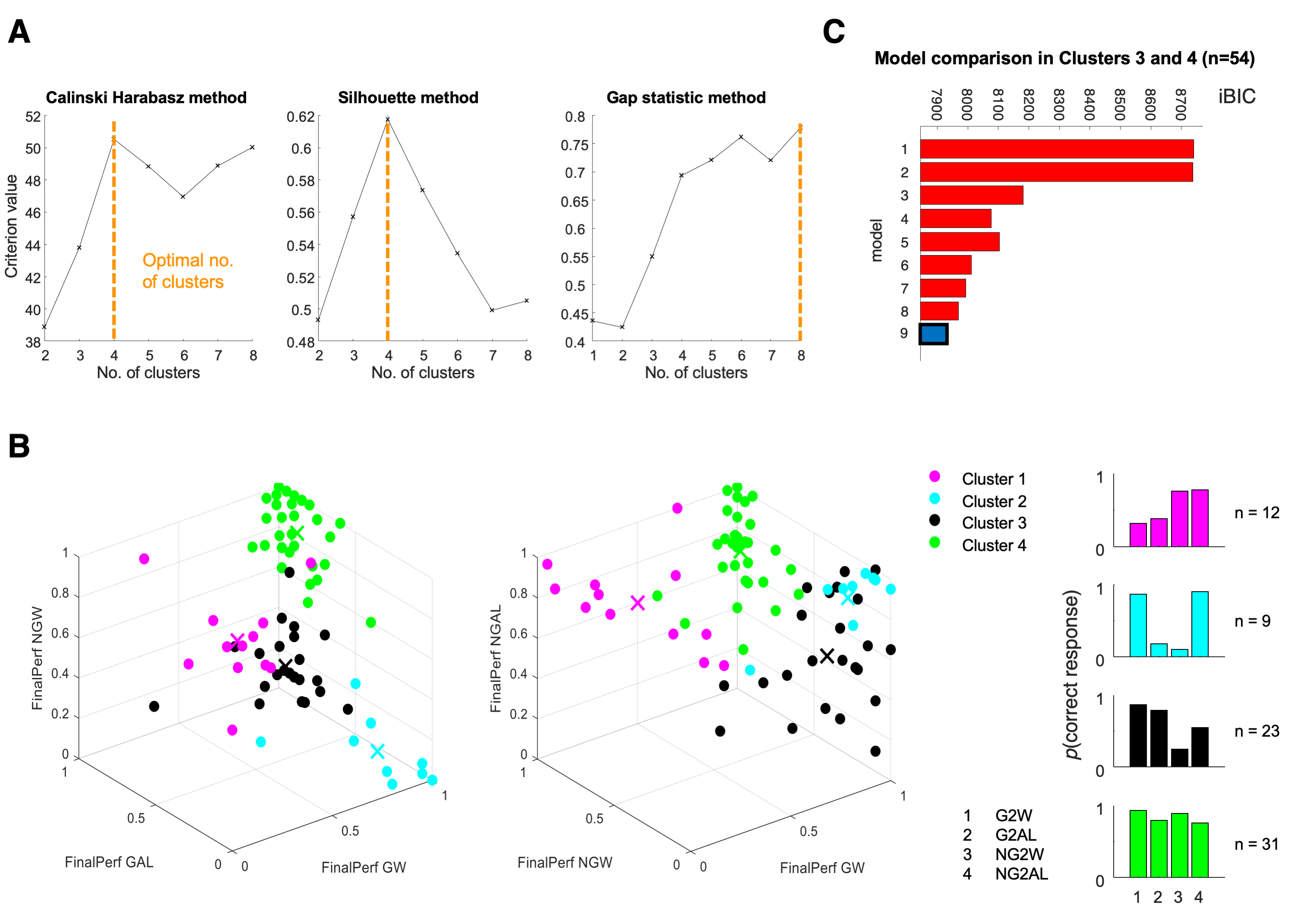

Supplement: FigureS6_bhz327 [file figures6_bhz327.png]

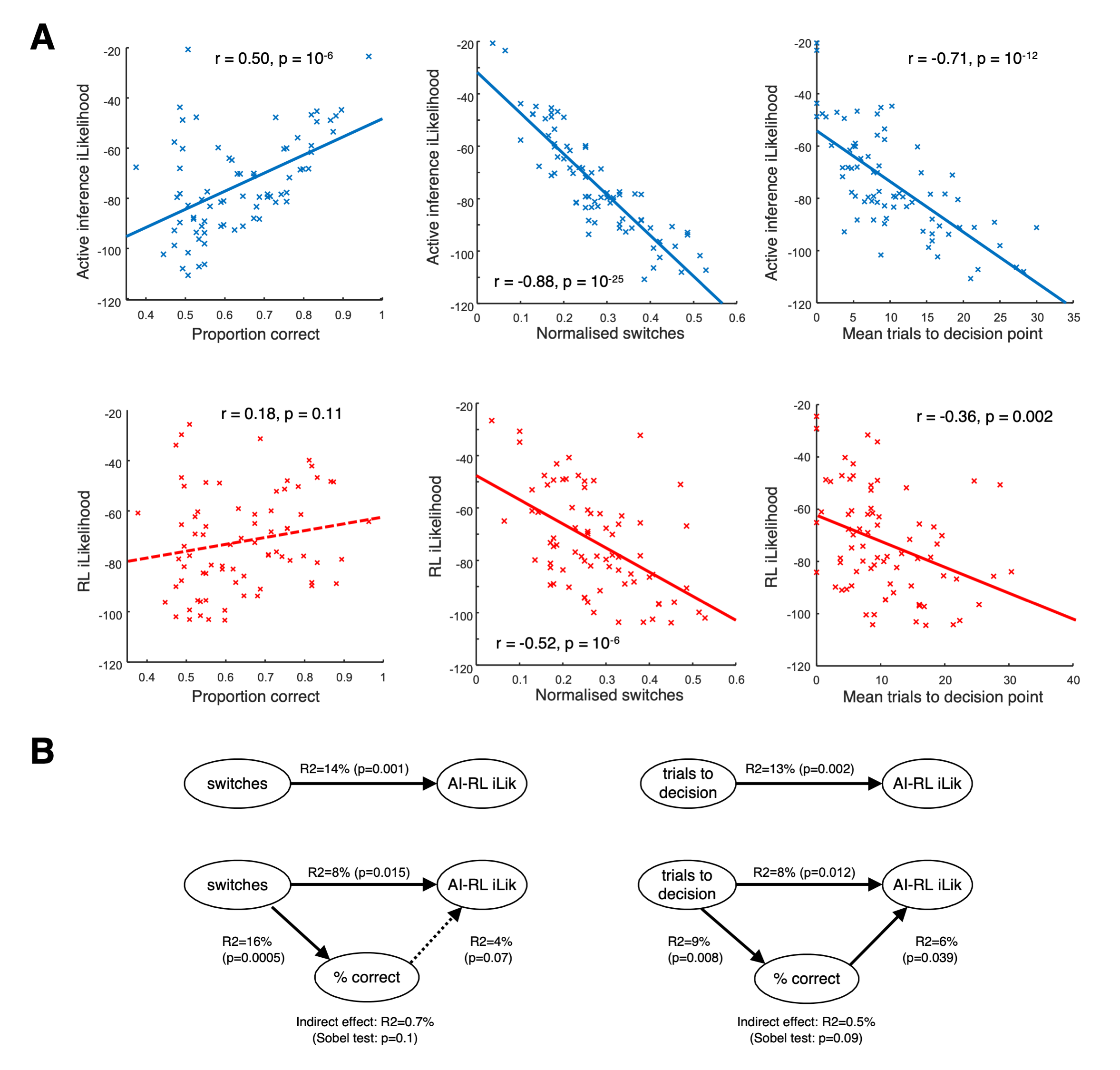

Supplement: FigureS7_bhz327 [file figures7_bhz327.png]

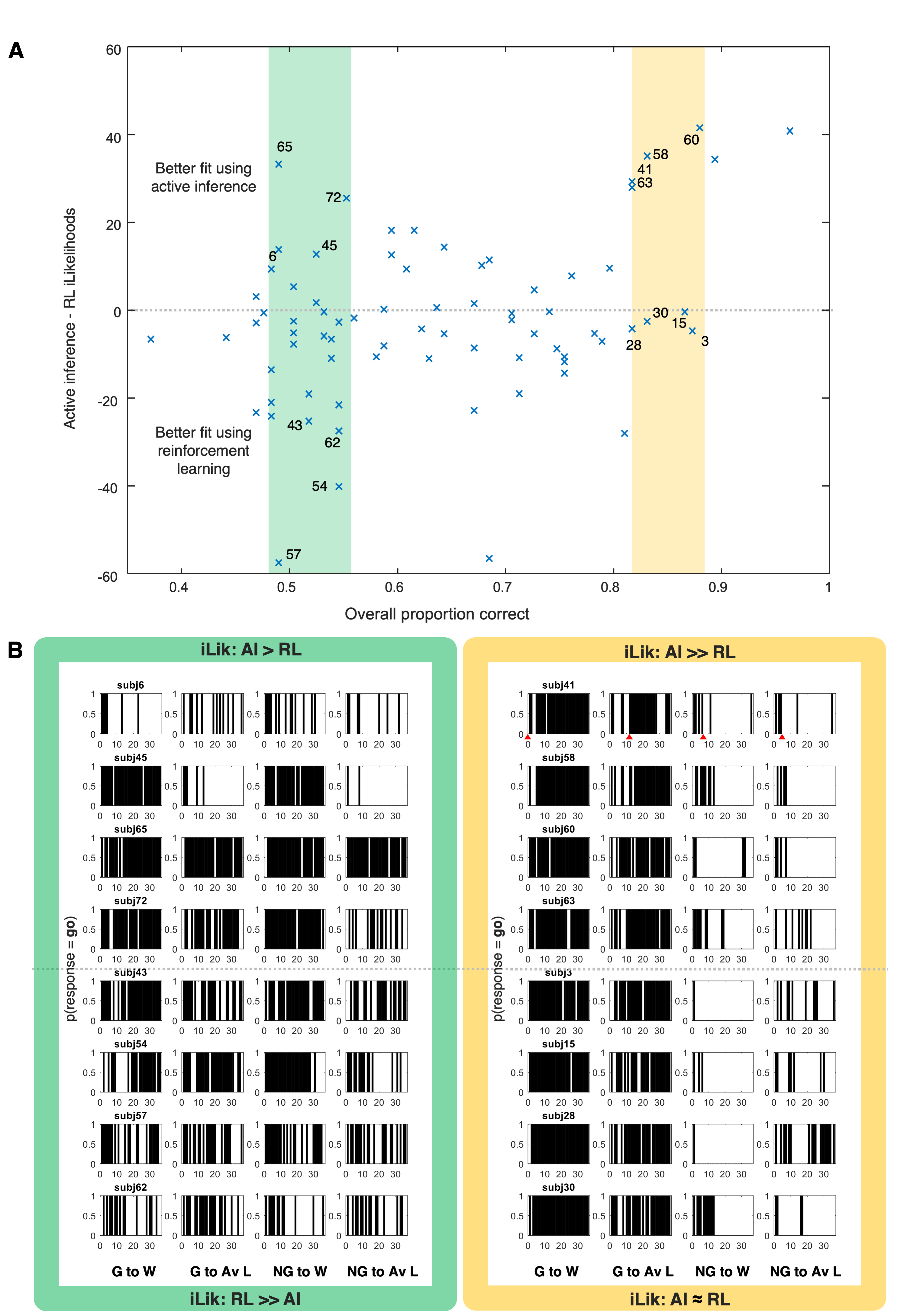

Supplement: FigureS8_bhz327 [file figures8_bhz327.png]

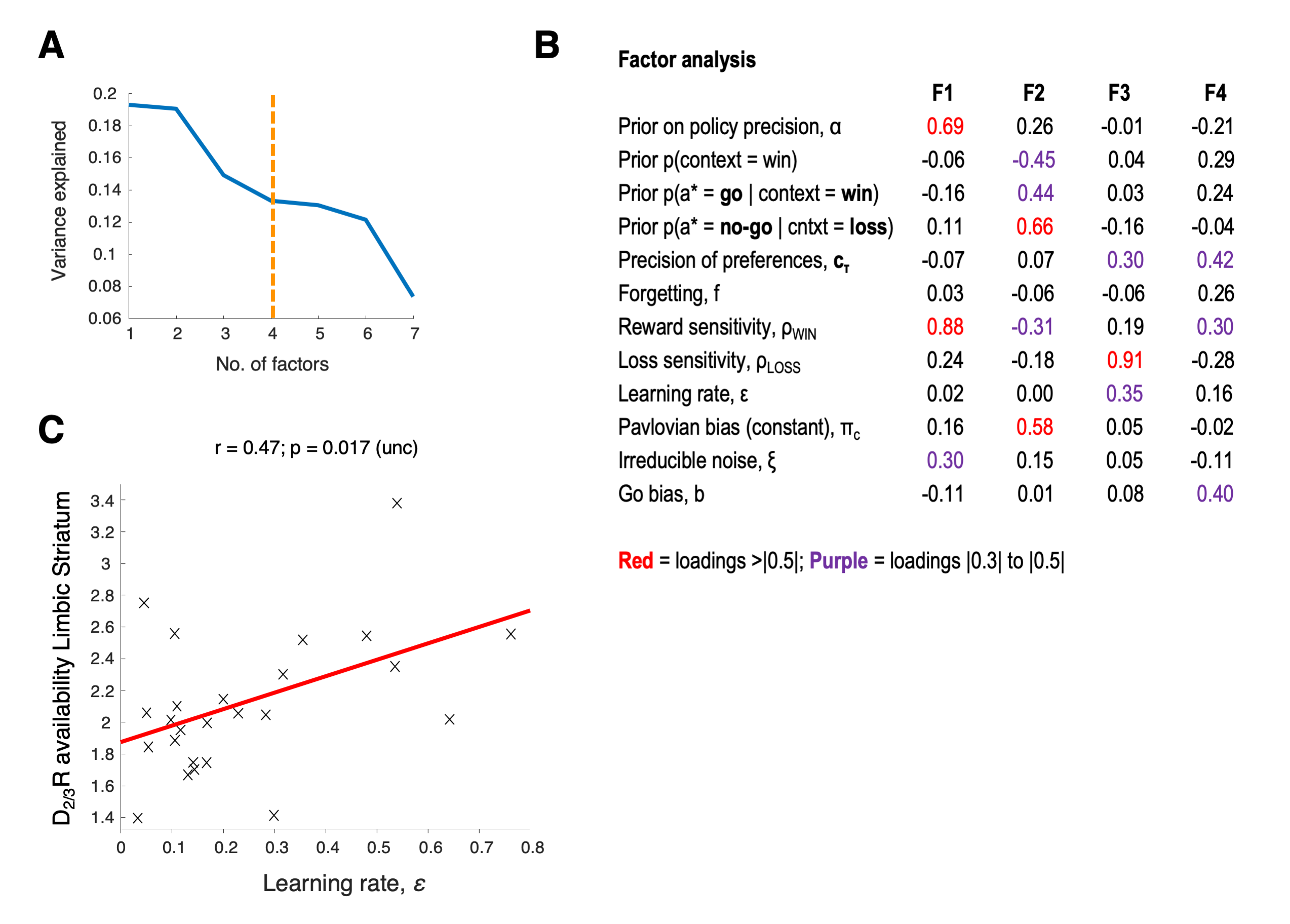

Supplement: FigureS9_bhz327 [file figures9_bhz327.png]
