# Supplementary material for: Variability in Action Selection Relates to Striatal Dopamine 2/3 Receptor Availability in Humans: A PET Neuroimaging Study Using Reinforcement Learning and Active Inference Models
Source: Cereb Cortex. 2020 Feb 21;30(6):3573–89. doi: 10.1093/cercor/bhz327 (PMC7233027; doi:10.1093/cercor/bhz327)
Supplement: Supplement_v4_bhz327 [file supplement_v4_bhz327.docx]

# Supplementary Information for

Variability in action selection relates to striatal dopamine 2/3 receptor availability in humans: a PET neuroimaging study using reinforcement learning and active inference models

Rick A Adams^1,2,3,4^†, Michael Moutoussis^5,6^, Matthew M Nour^3,4,7^, Tarik Dahoun^3,4,8^, Declan Lewis^1^, Benjamin Illingworth^1^, Mattia Veronese^9^, Christoph Mathys^6,10,11^, Lieke de Boer^12^, Marc Guitart-Masip^6,12^, Karl J Friston^5^, Oliver D Howes*^3,4,7^, Jonathan P Roiser*^1^

^1^Institute of Cognitive Neuroscience, University College London, 17 Queen Square, London, WC1N 3AZ, UK

^2^Division of Psychiatry, University College London, 149 Tottenham Court Road, London, W1T 7NF, UK

^3^Psychiatric Imaging Group, Robert Steiner MRI Unit, MRC London Institute of Medical Sciences, Hammersmith Hospital, London, W12 0NN, UK

^4^Institute of Clinical Sciences, Faculty of Medicine, Imperial College London, Hammersmith Hospital, London, W12 0NN, UK

^5^Wellcome Centre for Human Neuroimaging, University College London, 12 Queen Square, London, WC1N 3BG, UK

^6^Max Planck-UCL Centre for Computational Psychiatry and Ageing Research, 10-12 Russell Square, London, WC1B 5EH, UK

^7^Department of Psychosis Studies, Institute of Psychiatry, Psychology & Neuroscience (IoPPN), King’s College London, London, SE5 8AF, UK

^8^Department of Psychiatry, University of Oxford, Warneford Hospital, Oxford, UK

^9^Centre for Neuroimaging Sciences, Institute of Psychiatry, Psychology & Neuroscience (IoPPN), King's College London, London, SE5 8AF, UK

^10^Scuola Internazionale Superiore di Studi Avanzati (SISSA), Via Bonomea 265, 34136 Trieste, Italy

^11^Translational Neuromodeling Unit (TNU), Institute for Biomedical Engineering, University of Zurich and ETH Zurich, Wilfriedstrasse 6, 8032 Zurich, Switzerland

^12^Aging Research Center, Karolinska Institute, 171 65 Stockholm, Sweden

*joint senior authors

†corresponding author:

Dr Rick Adams, Institute of Cognitive Neuroscience, University College London, 17 Queen Square, London, WC1N 3AZ, UK

[rick.adams@ucl.ac.uk](mailto:rick.adams@ucl.ac.uk)

# Supplementary Methods

## Behavioural task

The instructions to participants were as follows:

“In this task you will see one of four different pictures on screen in every trial. You must work out the best action to take after seeing each picture, by trial and error. The only actions you can take are to press the space bar, or do nothing. You must make your action when you see the white circle: you have half a second to decide what to do after it appears, so you must respond quickly. After you choose to press or not press, you will get feedback. After some pictures you can win 10 pence (a green upward arrow) or get nothing (a yellow bar), and in other pictures you can lose 10 pence (a red downward arrow) or get nothing (a yellow bar). If you choose the best action for a given picture, you will get the best feedback available 80% of the time, and the worst feedback 20% of the time, and vice versa for the worst action. The best action to take for every picture will not change throughout the whole task. The pictures will be presented in a random order. We will begin with a practice session during which you will get used to how much time you have to respond after seeing the circle.”

Participants were also asked if they wanted to clarify any aspect of the instructions.

## Computational modelling – active inference

To illustrate the role of policy precision $\gamma$ and its prior, $\alpha$, two simulations of the active inference agent performing the task in both easy (G2W) and difficult (NG2W) contexts, and using parameter values typical of those subjects scoring <70% (Figure S3A&B) and >70% (Figure S3C&D) in the last 20 trials were performed. As each agent becomes more confident about the context it is in, policy precision $\gamma$ rises to an upper bound set by $\alpha$. $\gamma$ also increases and decreases in response to desired or undesired outcomes (respectively), making action selection less deterministic following an undesired outcome even if beliefs about the context don’t change. Actions have greater consistency in the agent with higher $\alpha$ (Figure S3C&D: see the legend for more details).

We performed parameter recovery analyses to ensure that we could reliably recover parameters of interest. The key parameters for the analyses presented here are AI parameter $\alpha$ (Model 9), and RL parameters $\rho_{WIN}$, $\rho_{LOSS}$, $\xi$ and $\varepsilon$ (Model 7). For each parameter, we simulated 200 datasets using the mean values of all parameters except the parameter of interest, whose 200 different values were evenly spaced from the minimum to the maximum values in our subjects’ data. We then estimated the parameters from this simulated data, and assessed the correlations between the varying parameter generating the data and its values estimated from the data. It can be seen that most are very reliably estimated (Figure S4A): only irreducible noise $\xi$ is less well recovered (*r* = 0.51). The correlation for $\rho_{LOSS}$ is not shown: it was *r* = 0.82. Furthermore, in their recovery analyses, $\alpha$, $\rho_{WIN}$, and $\xi$ did not correlate at *r* > 0.3 with any other parameter, except for $\alpha$ correlating with $c_{\tau}$ at *r* = 0.77 (hence $c_{\tau}$ was virtually fixed in our subsequent parameter estimation). We can therefore be confident that the relationships between these decision stochasticity parameters and striatal D_2/3_R availability in Figure 5 are reliable and not due to hidden trade-offs with other parameters.

## Factor analysis

We were interested in whether a single latent variable could account for much of the variance in the different parameters explaining various aspects of ‘choice stochasticity’ in the AI and RL models (i.e. $\alpha$, $\rho_{WIN}$, $\rho_{LOSS}$, and $\xi$). As neither model was likely to be perfect, and as some subjects were better fit by AI and some by RL, we reasoned that a latent choice stochasticity variable might show a stronger relationship with striatal D_2/3_R availability than any single parameter. We therefore performed a factor analysis (FA): this method finds latent variables (factors) that explain more variance than is explained by any single variable in the data. It is similar to principal component analysis (PCA) but with some important differences: i) FA assumes that the total variance in the data consists of both common variance (shared across variables) and unique variance, which includes measurement error, whereas PCA assumes all variance is common, thus its components are more likely to contain error variance; ii) PCA tries to explain the most possible variance with each successive component, whereas FA tries to distribute the variance explained more evenly amongst the factors. It does this using rotation methods, which try to ensure that each item loads most on a single factor, and that each factor has high loadings for only a subset of items. A typical approach to FA is to perform an initial exploratory analysis to assess how much variance is explained by successive factors (plotted in a ‘scree’ plot), and to use the number of factors corresponding to the point of this plot where the amount of variance explained per factor begins to level off (the ‘elbow’). The FA is then repeated using this number of factors. In our FA, we found little benefit of adding extra factors beyond four (Figure S9A), and so used four factors in the final FA (Figure S9B). The loadings of each item are effectively correlations of that item with the underlying factor: those above 0.3 can be regarded as meaningful. The first factor is clearly a ‘choice stochasticity’ factor, as three of the four relevant parameters load on it: two very strongly ($\alpha$ and $\rho_{WIN}$).

# Supplementary Results

## Computational modelling results

The subjects who underwent PET scanning had almost identical behavioural and modelling results to the wider sample. In Figure S5 their proportions of correct responses, model comparison results, and mean responses per condition (along with model simulations) are plotted in the same format as Figure 3. The winning model is slightly different: Model 6 wins here (it was a narrow second in the full sample): it has an adaptive Pavlovian bias rather than a fixed one, but is otherwise identical.

## PET results

We found that the ‘choice stochasticity’ factor derived from the factor analysis across parameters from the AI and RL models – on which $\alpha$, $\rho_{WIN}$ and $\xi$ all loaded – also had linear and quadratic relationships with D_2/3_R availability in the associative striatum (overall F(2,22) = 6.7, *p* = 0.005, R^2^(adj) = 0.32; linear *p* = 0.005, quadratic *p* = 0.009) and across the striatum as a whole (overall F(2,22) = 7.3, *p* = 0.004, R^2^(adj) = 0.34; linear *p* = 0.003, quadratic *p* = 0.009) but not within the sensorimotor striatum (overall F(2,22) = 3.0, *p* = 0.07). No other factors correlated with limbic striatal D_2/3_R availability (all p > 0.2).

We found relationships we had not hypothesized between model parameters and limbic striatal D_2/3_R availability. There was a linear correlation with RL learning rate $\varepsilon$ (*r* = 0.47, *p* = 0.017; Figure 6A), but this was not significant following multiple comparisons correction. We also detected relationships between limbic striatal D_2/3_R availability and the AI priors $p(a^{*}=go|context=W)$ and $p(a^{*}=nogo|context=\mathrm{AL})$, but parameter recovery analysis indicated these parameters were not reliably estimated, so we don’t describe them further. No other parameters had significant (uncorrected) correlations with limbic striatal D_2/3_R availability.

# Supplementary Figures

**Figure S1: The active inference and reinforcement learning frameworks**

A – The general form of the active inference model for Markov Decision Processes, illustrated in terms of its probabilistic relationships. The variables include observable outcomes $\tilde{o}$, hidden states $\tilde{s}$, control states $\tilde{u}$, actions $\tilde{a}$, and precision over policies $\gamma$. The ~ notation denotes collections of variables over time, $\tilde{o}=\{o_{0},\ldots,o_{T}\}$. $\gamma$ has a gamma distribution with shape prior $\alpha$ and rate prior $\beta$*;* here $\beta$ is fixed to 1. Please see the Methods for the definition of $\mathbf{Q}$ and Figure S2 for illustrations of $\mathbf{c}$, $\mathbf{B}$ and $\gamma$ (the likelihood matrix $\mathbf{A}$ is not used in this task). The blue shaded quantities have to be inferred.


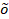

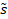

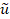

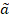

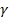

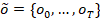

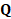

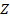


B – The variational update equations for states $s$, policies $\pi$ and precision over policies $\gamma$ in active inference for MDPs. The curved hat notation e.g. denotes sufficient statistics, and $\sigma$ denotes a softmax function. Note that $\gamma$ goes up when a policy is likely to get the agent to its goal (i.e. when $\mathbf{Q}$ is less negative). As $\beta$ (the gamma distribution rate parameter) was fixed to 1, the prior $\alpha$ effectively prescribes the maximum value $\gamma$ can take (see Figure S2E). In the simplified schematic on the right, the variables are overlaid on brain areas that are hypothesised to encode them (although clearly some will be much more widespread, e.g. the encoding of states).


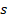

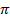

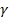

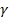

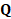

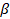

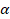

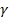


C – The RL model equations for Model 7, the winning model across all subjects. The fifth equation depicts the method of computing $W_{t}(a,s)$ used by the models containing the constant Pavlovian bias, $\pi_{c}$ (see Methods): $\mathrm{sgn}(V\left( s \right))$ denotes the signum function, which is 1 if $V\left( s \right)>0$, –1 if $V\left( s \right)<0$, and 0 if $V\left( s \right)=0$.

**Figure S2: The active inference model for the go no-go task**

A – The priors and parameters used in this figure: they are slightly exaggerated for illustrative purposes. The lower part of this section shows how the agent’s belief distribution $D(\mathrm{ctxt}_{t=0})$ about the prevalence of the four contexts is derived from its Pavlovian priors (the 2^nd^ to 4^th^ in the list). This distribution is plotted on the right.

B – The agent’s preferences about states $\sigma(r(s_{T});c_{\tau})$ are shown in the matrix: the columns are contexts, the rows are observations (initial, lose, null, win): $o=\left\{ init,-1,0,1 \right\}$. A precision of prior preferences $c_{\tau}$ of 3 means the agent prefers the better to the worse outcome available by a factor of exp(3), roughly 20 (we use $c_{\tau}=3$ for illustrative purposes; participants’ estimated values were much higher). In this model, the agent’s prior preferences $c$ are the product of its preferences about states and how likely those states are, i.e. $D(\mathrm{ctxt}_{t=0})$. Impossible outcome states (e.g. staying in the initial state) have values of 0. This vector is plotted on the right.


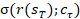

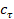

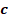

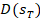

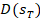


C – The $B$ matrices determine the possible transitions in states given the agent’s control states $u=\{Go,NoGo\}$. Here the transition matrix for the action *go* is shown. It maps from the columns to the rows: the blue arrow shows that choosing a *go* action in the ‘*go* to win’ context’s initial state maps to a *win* outcome with 80% probability. Actions have no effect on outcomes states, which map on to themselves.


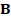


D – This illustrates the effect of the forgetting parameter $f$ on the belief distribution $D(\mathrm{ctxt}_{t})$ about which state a given context corresponds to, given the previous outcome. Here, the agent has performed ‘no go’ and received a reward, so it believes it is 80% likely to be in the NG2W context, and 20% G2W (at $n_{gap}=0$) . If this context is not experienced for $n_{gap}$ trials (≤3 are shown here), the agent’s belief distribution $D(\mathrm{ctxt}_{t-ngap})$ slowly decays to $D(\mathrm{ctxt}_{t})$, resembling its prior beliefs $D(\mathrm{ctxt}_{t=0})$. In the plotted example, the agent believes this context is most likely NG2W, but over the next 3 trials, the context isn’t experienced, and this belief distribution returns towards its priors. When the context is experienced again, the agent’s decayed belief about the context given the previous outcome ($D(\mathrm{ctxt}_{t})$) is incorporated into its posterior beliefs about that context, which do not decay. Note that each context has its own belief distribution and that they are independent in this model.


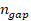

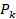

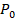


E – This illustrates the effects of outcomes on policy precision $\gamma$, and the effects of $\gamma$ on action selection. The equations (from Equation 5) show how the quality of policies $\mathbf{Q}$ is computed (first line) and that the probability the agent chooses the best policy in a given state is a function of how likely that policy is to achieve its goals ($\mathbf{Q}$), $\gamma$ and normalizing constant $Z_{\pi}$ (second line). The plot on the right shows that as $\gamma$ increases, the choice of the best action (*go*) becomes more deterministic, just like a softmax temperature parameter (Figure 2A). However, the plot on the left shows that $\gamma$ is also updated from trial to trial (according to Equation 6): the increases and decreases correspond to trials with good or bad outcomes respectively.


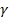

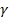

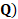

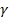

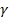

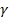

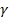

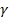


**Figure S3: Simulations using active inference (moderate and high performing subjects)**

This figure shows the beliefs, events and precision updates for two simulated agents each performing two conditions of the task: *go* to win (A & C) and the more difficult *no-go* to win (B & D). The moderately performing (A&B) agent’s parameter values were representative of the subjects scoring <70% in the last 20 trials: $\alpha$ = 1.5, $p\left( \mathrm{context}=W \right)$ = 0.53, $p\left( a^{*}=go | \mathrm{context}=W \right)$ = 0.53, $p(a^{*}=nogo|context=\mathrm{AL})$ = 0.51, $c_{\tau}$ = 5.8, $f$ = 0.6. The high performing (C&D) agent’s parameter values were representative of the subjects scoring >70% in the last 20 trials: $\alpha$ = 2, $p\left( \mathrm{context}=W \right)$ = 0.51,$p\left( a^{*}=go | \mathrm{context}=W \right)$ = 0.50, $p(a^{*}=nogo|context=\mathrm{AL})$ = 0.50, $c_{\tau}$ = 5.8, $f$ = 0.5.

The upper rows show beliefs about which context the agent is in and which control state is appropriate for this context. The middle rows show the actions taken (in white) and the outcomes (+1 is win, 0 is no-change, -1 is lose). The bottom rows show the updates to policy precision $\gamma$: when the agent is confident about the state it is in and the appropriate action to take (upper row), and when the outcomes it desires occur (middle row), $\gamma$ reaches the maximum value, set by its prior $\alpha$.

A – Despite believing *go* to win to be the likeliest context at the start, in the *go* to win context it chooses *no-go* and obtains a (low probability) reward in the first trial, so it takes almost 10 trials to become confident the correct action is actually *go*. Once it does so, it chooses *go* consistently, even during a sequence of unlucky outcomes (trials 17-23) when it doesn’t receive the reward despite choosing the correct action four times. Note that once it is confident about the context and control states, unlucky outcomes have little impact on these beliefs, but they do impact on policy precision. The unlucky outcome in trial 34 causes policy precision to fall and the agent to choose *no-go* on trial 35, despite its strong belief that the context is *go* to win.

B – The agent believes *no-go* to win to be less prevalent, so it takes longer to become confident that this is the context. Interestingly, between trials 9-12, despite choosing to *no-go* and being rewarded each time, the agent’s belief that the context is *go* to win increases. This is because of its prior belief that the *go* to win context is more prevalent. Once the agent is confident the context is *no-go* to win, however, it chooses *no-go* consistently.

C – Because this agent’s prior on policy precision $\alpha$ is higher than the previous agent’s, it chooses actions more deterministically. Nevertheless, it is not perfectly deterministic: its sole decision to *no-go* is a chance one made when its policy precision is high (trial 31).

D – Three consecutive unlucky outcomes in trials 15-17 make the agent revise its belief that the context is *no-go* to win in favour of *go* to win, so this agent takes longer than the agent in Figure S3A&B to become confident this state is *no-go* to win. Despite this, its greater policy precision means it chooses more correct actions in this state (29) than the other agent (26).

**Figure S4: Parameter recovery analyses & performance in the last 20 trials for each participant.**

A – Parameter recovery was assessed by simulating 200 datasets using the mean values of all parameters except the parameter of interest, which was scaled between its minimum and maximum values in the empirical dataset. This was repeated for each parameter of interest in AI ($\alpha$, Model 9), and in RL ($\rho_{WIN}$, $\rho_{LOSS}$, $\xi$ and $\varepsilon$, Model 7). Parameters of interest were those showing relationships with D_2/3_R availability (or a lack of one, in the case of $\rho_{LOSS}$). Correlations between the generating values and those estimated from the data are shown here ($\rho_{LOSS}$ is omitted but had *r* = 0.82).

B – These bar plots show the probability each participant (n = 75) made the correct response in the last 20 trials of each condition. Note that each condition – *no-go* to win in particular – has numerous participants that are performing well below chance level (0.5) – implying they have formed the wrong inference about that condition, rather than no inference at all.

**Figure S5: Behaviour and model comparison results in only the subjects who had PET scans (n = 25).**

This figure shows the average performance in each condition (A), model comparison results (B) and mean and model belief trajectories (C) in the same format as Figure 3, for the subset of subjects who underwent PET scanning. It can be seen that this subgroup are representative of the whole sample (Figure 3), in that their results are almost the same: one difference is that Model 6 narrowly beats Model 7 here, rather than the other way around. Active inference (Model 9) remains in 5^th^ place.

**Figure S6: K-means clustering analysis of behaviour and cluster-based model comparison**

Rather than choosing a somewhat arbitrary threshold for good performance (above chance, i.e. >50%, in the last 20 trials of each condition), we tested whether a data-driven method could detect a subgroup or subgroups of participants who were better fit by active inference. We used *k*-means clustering to do this: this method partitions observations into *k* clusters in which each observation belongs to the cluster with the nearest mean, and the within-cluster sum of squares (i.e. variance) is minimised (equivalent to maximizing the between cluster sum of squares). Prior to clustering, a preferred number of clusters *k* must be determined – there are numerous methods to do this.

A – Three standard methods for selecting an optimal number of clusters *k* were performed: in each, the optimal *k* produces the highest index value (shown with a dashed orange line). The Calinski-Harabasz index (left plot) evaluates cluster validity based on the average within- and between-cluster sum of squares. The Silhouette index evaluates cluster performance based on the pairwise difference of within- and between-cluster distances (Liu et al., 2010). The gap statistic compares the within-cluster sum of squares for different *k* to its expectation under an appropriate null reference distribution (Tibshirani et al., 2001). We clustered participants on the basis of their average proportion of correct responses in each condition across the last 20 trials. We used the last 20 trials rather than all trials because one would expect the former to highlight individual differences in performance. The first two of these methods identified four as the optimal number of clusters, so we used *k* = 4.

B – The four clusters are illustrated. Each scatter plot shows the average proportion of correct responses in three of the four conditions for each participant. The dots are coloured according to cluster assignments. The centroids of each cluster are indicated with an X, and plotted in the bar graphs on the right (in the same format as Figure 3A). Cluster 1 participants (n=12) prefer *no-go* to *go* and were best fit by Model 4, in which outcome sensitivity doesn’t differentiate between rewards and punishments. Cluster 2 participants (n=9) are very Pavlovian and were best fit by Model 6, in which state values have maximum influence over Pavlovian responding. Cluster 3 participants (n=23) prefer *go* to *no-go* and were best fit by Model 9 (active inference). Cluster 4 participants (n=31) are high-performing and were also best fit by Model 9. C – The results of model comparison performed on the largest groups together (3 & 4): the active inference model wins (iBIC margin = 40). It also wins in clusters 3 and 4 individually, but is 5^th^ in cluster 2 and 7^th^ in cluster 1 (not shown). Models are listed in the same order as in Figures 3 and 4.

**Figure S7 – Relationships between integrated likelihoods and aspects of performance**

A – This figure shows the relationships between the integrated likelihoods in AI (top) or the winning RL Model 7 (bottom) and the number of response switches (normalised to between 0 and 1) or mean trials to the ‘decision point’. It is clear that although both models show significant relationships between these variables, the AI model has much stronger correlations between both consistency (Steiger’s *Z*, *p* = 10^-6^) & speed of deciding (Steiger’s *Z*, *p* = 0.00004) and model likelihood. This is striking because neither are necessarily indications the participant is performing well – the participant could also be quickly and consistently wrong.

B – Mediation analyses showing that both the number of response switches (left) and the number of trials to the ‘decision point’ (right) contribute to active inference’s improvement in performance over the RL model independently of accuracy (the mediating variable in each case being % correct). Put simply, making consistent responses and deciding early explain being better fit by AI than RL independent of whether these responses were actually correct.

**Figure S8 – Comparing participants of similar performance who are fit best by different models**

A – An enlarged version of Figure 4A, showing the relationship between the overall proportion correct and the difference between AI and RL likelihoods. The numbers are participants whose responses are shown in the following figure. The bands of colours correspond to participants performing at different levels. We sought to compare participants whose performance is roughly the same overall, in terms of proportion correct, but who show the largest possible discrepancies in their likelihoods for the active inference and RL models.

B – Why are some participants best fit by either AI or RL? The responses of individual participants in each of the four conditions are plotted on each row. The upper four rows show participants who are best fit by AI, the lower four rows show participants who are best fit by RL. The colours correspond to the colours in A – orange and green are better and worse performing participants. The red arrows show the ‘decision points’ for participant 41, where s/he seems to change from consistently choosing one action to the other.

The main difference between the participants in the orange box is that the participants best fit by AI are more consistent. The lower four (best fit by Rescorla-Wagner RL) all revert to incorrect responses in the latter half of the *no-go* to avoid loss condition. In the worst-performing participants (dark green box) one can still see a tendency for the participants best fit by AI to be more consistent in their responding (even if they are completely incorrect in two conditions, as in participant 45 and participant 65), whereas the participants best fit by RL perform equally badly on average, but more stochastically from trial to trial.

**Figure S9 – Factor analysis of the AI and RL (Model 7) model parameters and the D_2/3_R availability and RL learning rate relationship**

A – An exploratory factor analysis using seven factors showed that the variance explained by the factors levelled off at factor 4 (orange dashed line), so four factors were chosen for the final factor analysis. This was performed using ‘factoran’ in Matlab (using all default options, including varimax rotation).

B – The loadings of all parameters on the four factors are shown, with higher loadings coloured for emphasis. Parameters loading on the first factor ($\alpha$, $\rho_{WIN}$ and $\xi$ – but not $\rho_{LOSS}$) all affect response stochasticity. The Pavlovian parameters in both models ($p\left( \mathrm{context}=W \right)$, $p\left( a^{*}=go | \mathrm{context}=W \right)$, $p(a^{*}=nogo|context=\mathrm{AL})$ and $\pi_{c}$) loaded on the second factor, and RL loss sensitivity $\rho_{LOSS}$ strongly loaded on the third factor. Note that the purpose of this factor analysis was to just to demonstrate the relationships between the two sets of model parameters and the possible reasons underlying these relationships. These results don’t imply that these factors generalise outside this task, as both sets of parameters were estimated from the same data.

C – There is a linear relationship between RL learning rate $\varepsilon$ and D_2/3_R availability (*r=*0.47, 95% CI [0.10 0.73], *p*=0.017), but this was not significant following correction for 12 comparisons (six parameters per task).

## References

Liu Y, Li Z, Xiong H, Gao X, Wu J. 2010. Understanding of Internal Clustering Validation MeasuresProceedings of the 2010 IEEE International Conference on Data Mining, ICDM ’10. Washington, DC, USA: IEEE Computer Society. pp. 911–916. doi:10.1109/ICDM.2010.35

Tibshirani R, Walther G, Hastie T. 2001. Estimating the number of clusters in a data set via the gap statistic. *J R Stat Soc Ser B Stat Methodol* **63**:411–423. doi:10.1111/1467-9868.00293
